# The WOMED model of benign thyroid disease: Acquired magnesium deficiency due to physical and psychological stressors relates to dysfunction of oxidative phosphorylation

**DOI:** 10.1016/j.bbacli.2014.11.002

**Published:** 2014-11-12

**Authors:** Roy Moncayo, Helga Moncayo

**Affiliations:** WOMED, Karl-Kapferer-Strasse 5, AT-6020 Innsbruck, Austria

**Keywords:** Magnesium, Selenium, Coenzyme Q10, Oxidative phosphorylation, Uncoupling

## Abstract

**Background:**

The aim of this study was to discern whether a relation between biochemical parameters, sonography and musculoskeletal data exists in cases of hyperthyroidism and whether they are modifiable through supplementation with selenomethionine and magnesium citrate as well as by acupuncture and manual medicine methods.

**Results:**

A direct correlation between whole blood selenium and serum magnesium was found in subjects without thyroid disease and in menopausal women while it was reversed in cases of thyroid diseases as well as in patients with depression, infection, and in infertile women. Vascularization indices were elevated in cases of newly diagnosed benign thyroid diseases. Musculoskeletal changes i.e. lateral tension and idiopathic moving toes, as well as situations of physical and psychological stress and minor trauma and infection led to an increase of vascularization. Magnesium levels correlated negatively with these two conditions. The supplementation brought a reduction of the vascularization indices and reduced the incidence of idiopathic moving toes. Treatment of lateral tension required manual medicine methods and acupuncture (gastrocnemius). A small subgroup of patients showed a further reduction of hyper-vascularization after receiving coenzyme Q10.

**Conclusions:**

We interpret the elevated thyroid vascularization and low magnesium levels as signs of an inflammatory process related to the musculoskeletal changes. Improvement of thyroid function and morphology can be achieved after correcting the influence of stressors together with the supplementation regime. We hypothesize that the central biochemical event in thyroid disease is that of an acquired, altered mitochondrial function due to deficiency of magnesium, selenium, and coenzyme Q10.

## Highlights

• Definition of normal levels of magnesium in normal subjects as > 0.95 mmol/l.
• Normalization of TSH levels after Mg supplementation.
• 3D-perfusion patterns of the thyroid are responsive to magnesium supplementation.
• Need to treat psychological and physical stress in order to reduce magnesium needs.
• Increased thyroid perfusion in the first days after in-vitro fertilization.

## Graphical abstract

**Figure:**
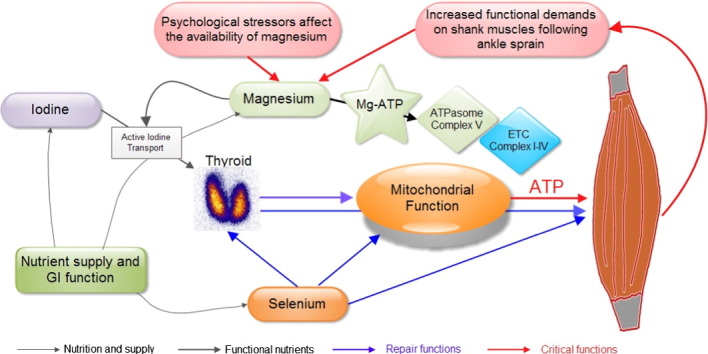

## 1 Introduction

The pathogenesis of benign thyroid diseases is a question that has puzzled medical science since the first clinical and pathological descriptions were made in the past centuries. Looking back into early moments of clinical research one can find the landmark descriptions on hyperthyroidism that were published between 1825 and 1840 by Parry, Graves, and von Basedow [1], [2], [3] while Hashimoto contributed with the histological picture of goitrous lymphocytic thyroiditis in 1912 [4]. In 1906 McCarrison described the beneficial use of iodine to treat endemic goiter [5]. Later investigations have confirmed the central role of iodine in maintaining normal thyroid function [6]. Since the 1950s the conception of “autoimmunity” has stepped into the description of potential mechanisms that affect thyroid function. An account of the development of this conception has been presented by Sawin. In his opinion: “autoimmune thyroid disease is now itself a major industry” (page 402 in [7]).

While iodine deficiency still prevails in some countries, benign thyroid disease is still being found in countries that have corrected this condition. Thus it is sensible to consider that additional factors can be involved in the pathogenesis of thyroid disease.

Since 2007 we have been engaged in research work centered on thyroid function and thyroid disease. These investigations had a starting point with patients presenting thyroid associated ophthalmopathy (TAO) [8], [9]. The key findings of these studies were the description of alterations of the musculoskeletal system including an inflammatory reaction in the shanks together with axial displacement, e.g. lateral tension [10], eccentric muscle action, the idiopathic moving toes (IMT) phenomenon, as well as an altered relationship between magnesium and calcium. Investigating the micronutrient status of these patients we found a high frequency of low levels of selenium in patients presenting either hypothyroidism or hyperthyroidism [11]. At the time this study on micronutrients was being carried out we started to supplement thyroid disease patients only with selenomethionine [12]. This procedure, however, did not lead to lasting results (unpublished data). In subsequent studies we have updated the reference values for thyroid function tests for the local iodine-sufficient population of Austria in different age groups [13], [14], [15]. In the field of sonography we have recently adapted a quantitative 3-dimension power Doppler method for the evaluation of thyroid vascularity [16]. This methodology was originally developed for gynecological examinations by Pairleitner et al. [17]. In 2014 we have been able to differentiate the clinical picture of so-called psychosomatic symptoms from hypothyroidism and found these symptoms to be directly associated with magnesium deficiency. In situations of increasing physical and/or psychological stressors patients show lower magnesium levels in blood [18]. In the discussion of these findings we hypothesized that low magnesium levels would alter the function of Complex V of oxidative phosphorylation knowing that magnesium is the central player in ATP synthesis through the F_1_F_O-_ATPase [19].

In this observational study we have now focused on patients with hyperthyroidism and thyroiditis applying the previously described methodology [18]. The merits of observational studies as stated by Black [20] were kept in mind in designing this study. Based on the frequent condition of low selenium and magnesium levels seen in patients with benign thyroid diseases the null hypothesis of this observational study stated that administering selenomethionine and magnesium citrate would neither affect thyroid function, nor vascularity, nor thyroid-associated symptoms. A second aim was to document thyroid vascularity parameters in a variety of situations that included mainly physical and psychological stressors and recording the changes achieved after supplementation.

## 2 Materials and methods

A consecutive series of 176 subjects, including 159 females, was included between 2011 and 2013 in this observational study. The age of the patients was 32.2 ± 11.1 years (mean ± S.D.), range 7 to 73 years. All subjects were examined and treated at our private institution (WOMED, Innsbruck, Austria). All procedures were done in accordance with the Declaration of Helsinki [21]. The term “The WOMED Model” refers to a combination of examination, diagnostic and therapeutical methods used routinely by us. This Model has been described and explained recently in a publication where we dealt with the topic of psychosomatics and hypothyroidism [18]. Data from this previous study (patients with hypothyroidism) was also included in the present one in order to compare them to hyperthyroidism.

The clinical examination was done as described before [8], [10], [18] where a central role is played by methods used in Applied Kinesiology (AK) [22], [23], [24]. An important addition to the previous approach is to look for any pelvic fault associated with respiratory action [22]. Postural changes that can be found through this examination include lateral tension and the idiopathic moving toes (IMT). Idiopathic moving toes are shown on the video file.

### 2.1 3-D quantitative power Doppler thyroid sonography

The 3-dimensional quantitative power Doppler sonography of the thyroid was done as described recently by us [16]. This examination delivers 3 parameters related to vascularization of the gland: 1) the vascularization index (VI) given as a percentage which reflects the number or density of vessels within the volume being examined, 2) the flow intensity of blood corpuscles during the 3D-sweep called flow index or FI, and 3) a combination of VI and FI which gives the vascularization flow index (VFI) thus reflecting a combination of the vessel density and the number of blood corpuscles [25], [26]). Ultrasound studies were done twice, at the initial and at the final visit.

### 2.2 Laboratory examinations

Thyroid function parameters, i.e. fT3, fT4 and TSH, as well as magnesium levels were recorded for all patients. The determinations were generally done at a local laboratory facility (Labor Philadelphy, Innsbruck, Austria, http://www.phillab.at/). Results from another laboratory which uses a similar methodology were also included. “Third party” results were excluded, i.e. a new test was ordered. Since not all thyroid antibody determinations came from the same laboratory, the results were simply classified as being either negative or positive according to the reference range of each method. Thyroid antibodies were not determined in follow-up examinations. In a later phase of the study we also included determinations of coenzyme Q10 (ubiquinone). We purposely chose these simple clinical chemistry tests because they can be easily carried out at any laboratory.

### 2.3 Stress evaluation

Based on the description made by Parry on the association of stress and fright with thyroid disease [1] we evaluated the presence of stressors in a simple declarative manner. The first question attempted to identify a previous traumatic situation in early life. Modern research uses the terms prenatal maternal stress [27], [28] and early life stress [29], [30] for these situations. The patients were asked in simple language whether they had had psychological or physical trauma as a child, care deprivation as a child, hospitalization without parental company, or if the mother had gone through a difficult pregnancy. The next 2 questions explored current situations such as daily hassles [31], [32] (at home or at work) or feelings of anxiety or fear, panic attacks, or irritability [33]. For subjects having a score of 2 or 3 a stress reduction treatment was done as described by us before [34]. Our original protocol has been extended to include acupoints found along the Triple Burner Meridian (TB) [18], i.e. acupoints located between TB4 (yáng chí) to TB8 (san yang luo) [35]. Physical stress in the form of competitive sport activity was also identified but was not included in the score. The patients were also asked to observe and report any changes of well-being and to recall if any slight traumatic events such as ankle sprain had occurred in the past years.

### 2.4 Linear regression studies for magnesium and selenium

Linear regression studies of magnesium and selenium levels were done using a historical set of data which had already been analyzed [11]. In this group of subjects selenium was measured in whole blood samples; magnesium was measured both in whole blood as well as in serum samples and compared to selenium levels. This set of patients included 15 controls (10 females), 51 menopausal women, 15 patients with thyroid disorders (14 females), 33 women with infertility, 17 patients with depression, and 25 patients with flu-like light respiratory infections.

### 2.5 Supplementation procedure

Based on the results of our previous investigations, a daily nutritional supplementation was designed to include selenomethionine (200 μg/d, Pure Encapsulations©, pro medico Handels GmbH, Graz, Austria) and a magistral formulation of magnesium citrate powder at a dose of 3 × 1.4 mmol/day. The choice of magnesium citrate is based on the beneficial effects in energetics as shown by Siekmeyer et al. [36] as well as on the positive effect on mitochondrial homeostasis [37]. Selenium was given daily for 4 weeks, afterwards only 3 times per week. Magnesium supplementation was given over a period of 12 weeks.

### 2.6 Weight distribution analysis

In a small series of 8 patients, body weight distribution was evaluated using a simple force plate system (Nintendo Wii) [38], [39].

### 2.7 Statistical analysis

Statistical analysis was done by RM using IBM SPSS Statistics 21.

## 3 Results

### 3.1 Analysis of magnesium levels according to sonography and laboratory results

**Fig. 1:**
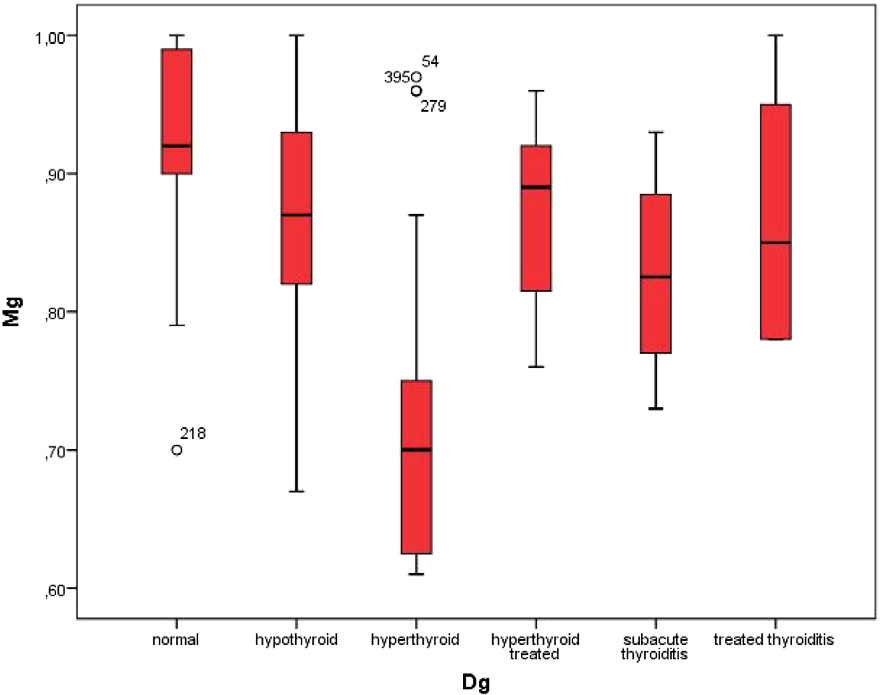
Serum magnesium levels in the study groups grouped according to thyroid function.

Based on the results of the ultrasound examination as well as on the thyroid function tests we classified 48 subjects as having both normal thyroid function tests as well as an uneventful morphology of the thyroid (controls), 89 cases as hypothyroid, 24 cases as hyperthyroid, and 4 cases as presenting sub-acute thyroiditis. Patients already being treated for thyroid dysfunction included 5 with hyperthyroidism, and 6 with thyroiditis. Based on this classification of thyroid function we found significant differences in the serum levels of magnesium, the highest levels were seen in the control group (mean 0.95 ± 0.08 mmol/l) and the lowest in patients with hyperthyroidism (mean 0.72 ± 0.14 mmol/l, p < 0.001). Fig. 1 depicts these differences.

### 3.2 Correlation studies for magnesium and selenium

**Fig. 2:**
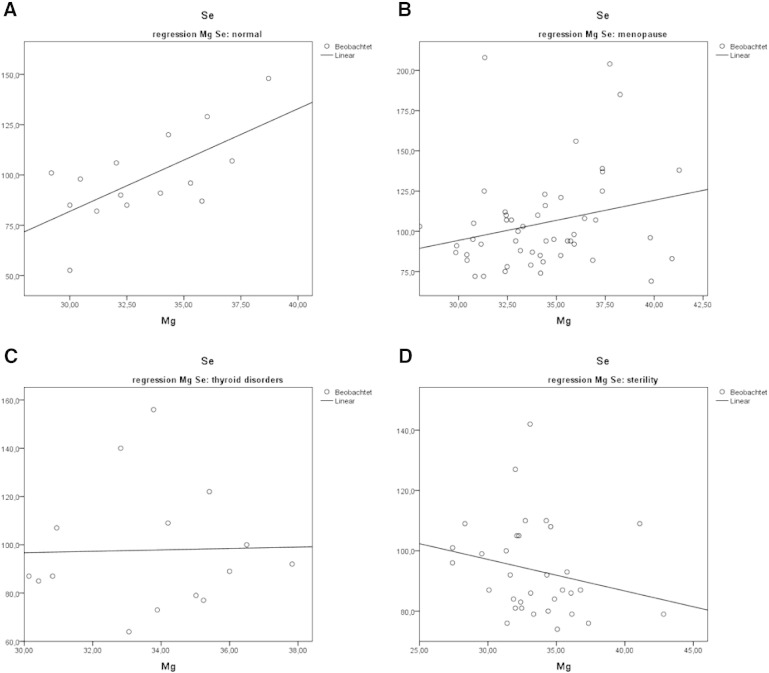
The panel shows the differences in the regression analyses between whole blood selenium and blood magnesium levels. A: normals. B: menopausal women. C: thyroid disorders. D: women with sterility. The measurement units are mg/l. Notice the change in the type of correlation in patients with thyroid disorders as well as in patient with sterility.

The correlation analyses of whole blood selenium and blood magnesium levels in the historical group showed a positive correlation in normal subjects as well as in menopausal women (Fig. 2, panels A–B). An inverse situation, i.e. a negative correlation, was seen in cases of infertility and in thyroid disorders (Fig. 2, panels C–D), in patients with depression, and in patients with light respiratory tract infections (data not depicted).

Interestingly, whole blood magnesium levels showed no correlation with serum levels of magnesium. Classifying the results of the whole blood magnesium determinations given as mmol/l into low (27.41–33.97), normal (34.02–36.00), and high (36.02–42.83) revealed the following ranges of serum levels of magnesium in mmol/l: 0.83–1.02, 0.86–1.00, and 0.60–1.00, respectively.

### 3.3 Analysis of the thyroid quantitative 3D-power Doppler sonography

**Fig. 3:**
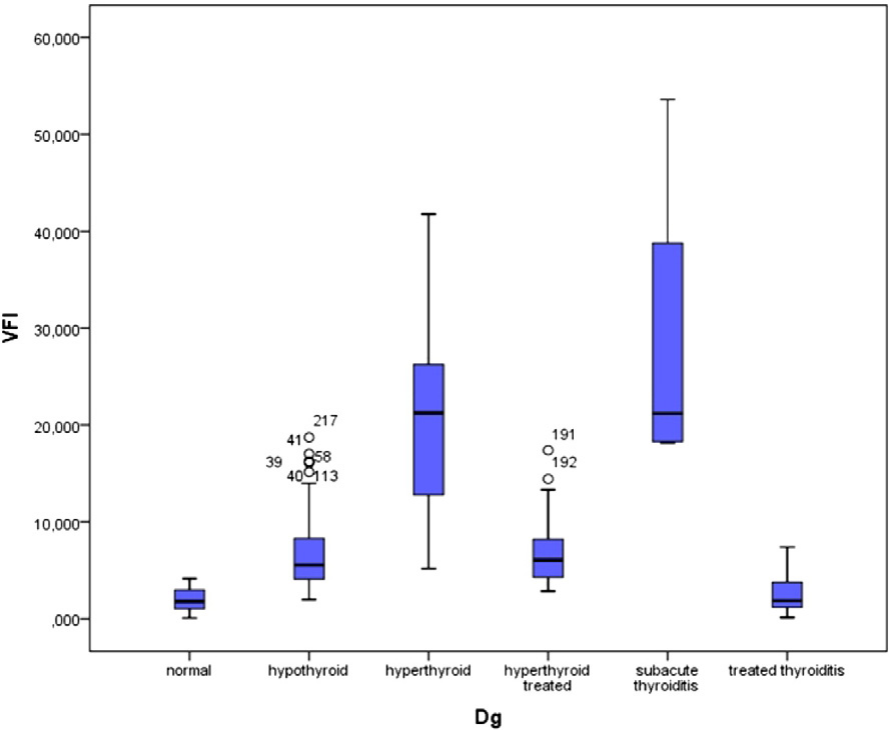
VFI values according to the clinical situation of thyroid disease.

The vascularization index (VI) given in % was found lowest in normal subjects (mean 5.56 ± 2.9%). Patients with a sub-acute thyroiditis presented initially high levels (mean 26.83 ± 16.6%) which dropped significantly after supplementation (mean 6.48 ± 1.8%). Patients with hypothyroidism presented high levels as compared to the controls (mean 15.41 ± 6.6%). The highest levels were seen in hyperthyroid patients (mean 42.29 ± 16.81%). Visual inspection of the 3D images allowed us to recognize the presence of rather thick vessels within the thyroid in some hyperthyroid patients having an elevated VI value. A persisting elevation of VI was seen in some hyperthyroid patients. The flow index showed less variation among the groups studied, however patients with hyperthyroidism as well as those with sub-acute thyroiditis had the highest values, i.e. 43.50 ± 5.14 and 41.78 ± 4.14, respectively vs. 34.99 ± 2.70 in the control group. The Vascularization Flow Index (VFI) showed the greatest differences among the groups. It was highest in hyperthyroid patients as well as in sub-acute thyroiditis. Hypothyroid patients as well as patients with treated thyroiditis showed intermediate high values. The values in the control group as well as in treated thyroiditis were similar (Fig. 3).

**Fig. 4:**
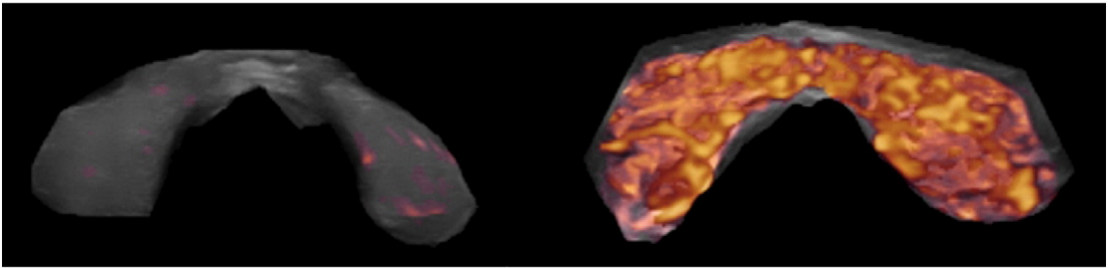
3-D reconstruction of a normal thyroid compared to the vascularity of a case of hyperthyroidism.

The 3D reconstruction of the power Doppler images (Fig. 4) clearly shows the differences of vascularization intensity between a normal control and an overt hyperthyroid case.

**Fig. 5:**
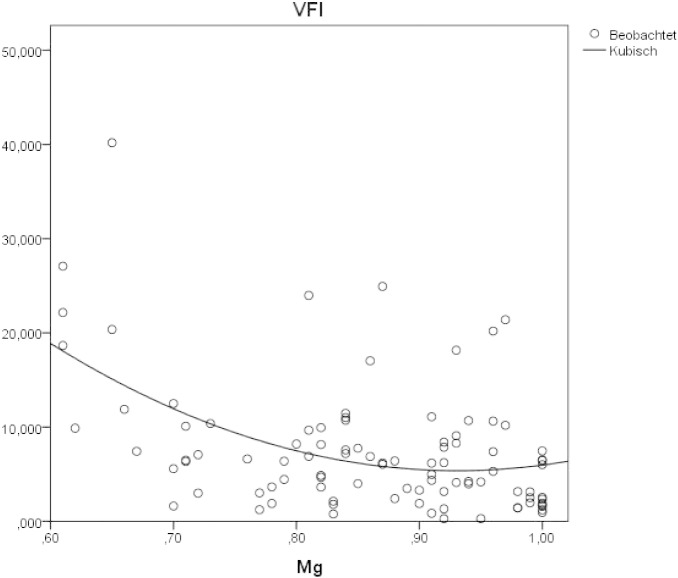
Relationship between magnesium levels and the thyroid perfusion index VFI.

A cubic regression curve with negative slope depicts the association between magnesium levels and the VFI values, i.e. low magnesium levels were associated with high VFI values (Fig. 5).

### 3.4 Thyroid perfusion parameters and thyroid serology

All three thyroid perfusion parameters were lower in patients with negative thyroid serology as compared to patients with positive thyroid serology (VI%: 10.7 ± 7.3 vs. 25.1 ± 16.6, FI: 36.8 ± 2.7 vs. 39.7 ± 3.4, VFI: 4.0 ± 2.8 vs. 10.5 ± 7.9). On the other hand, magnesium levels were higher in patients with negative serology (0.88 ± 0.08 vs. 0.82 ± 0.11 mmol/l).

### 3.5 Thyroid perfusion parameters in relation to the stress score

**Fig. 6:**
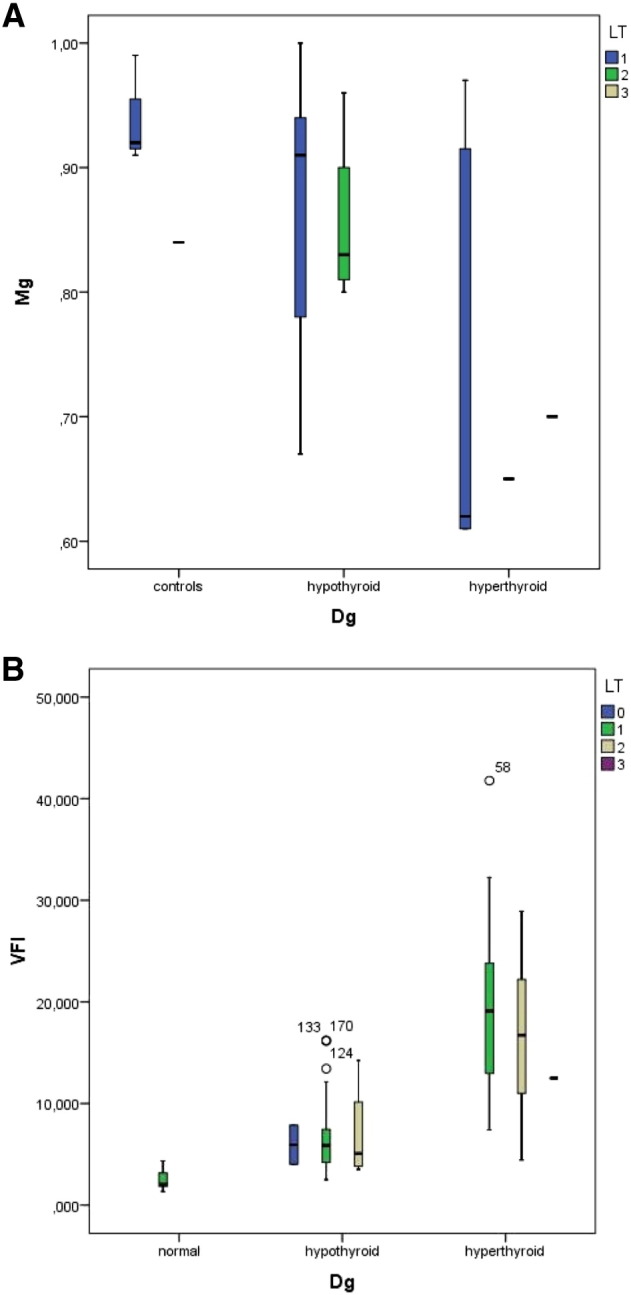
Reciprocal changes of magnesium levels and VFI in relation to findings of lateral tension.

The stress evaluation revealed a score of 1 in 27%, score of 2 in 40%, and score of 3 in 33% of patients. The VFI was directly correlated to the stress score such that the corresponding VFI values in stress scores 1 to 3 were 2.4 ± 1.6, 6.1 ± 5.1, and 11.2 ± 8.7, respectively. When the subjects were classified primarily by their thyroid function status, higher VFI levels were seen in hyperthyroid subjects and these values were also related to the presence of lateral tension, IMT, and the stress score. These relations are shown in Fig. 6A to B.

### 3.6 Influence of the supplementation procedure on TSH levels

In a group of 11 patients presenting elevated TSH levels although thyroid morphology on ultrasound was normal we provided only magnesium supplementation for 6 weeks. TSH levels dropped significantly (p = 0.022, paired *t*-test) afterwards. The observed TSH levels are shown on Table 1. Based on our current laboratory criteria for the interpretation of thyroid function tests [13], [14] the patients presented sub-clinical hypothyroidism at the initial examination. After supplementation the TSH levels were within the normal range (p < 0.02). Free T4 levels did not show a clear change tendency (p = 0.7).

### 3.7 Presence of musculoskeletal changes in patients with thyroid disease and effects of magnesium supplementation and manual therapy and acupuncture

The results of the clinical examination revealed lateral tension in the majority of patients presenting either hypothyroidism or hyperthyroidism (more than 90% of cases). Among the patients with lateral tension we found it to unilateral in 81.8%, and bilateral in 16.4% of cases. The phenomenon of IMT was seen in a smaller number of subjects (n = 46). In 60.4% of cases it was one-sided, in 31.25% of cases it was two-sided, and finally a complex situation was seen in 2% of cases. A complex situation means that IMT was elicited by rotation of the hand. One-sided lateral tension was observed in 26 cases presenting one-sided IMT, and in 12 cases presenting double-sided IMT. Bilateral lateral tension was seen in 3 cases with one-sided IMT, as well as in 3 cases with double-sided IMT. An example of IMT is shown in the accompanying video. The analysis of body weight distribution revealed a shift of 2–3% to the side not affected by lateral tension.

**Fig. 7:**
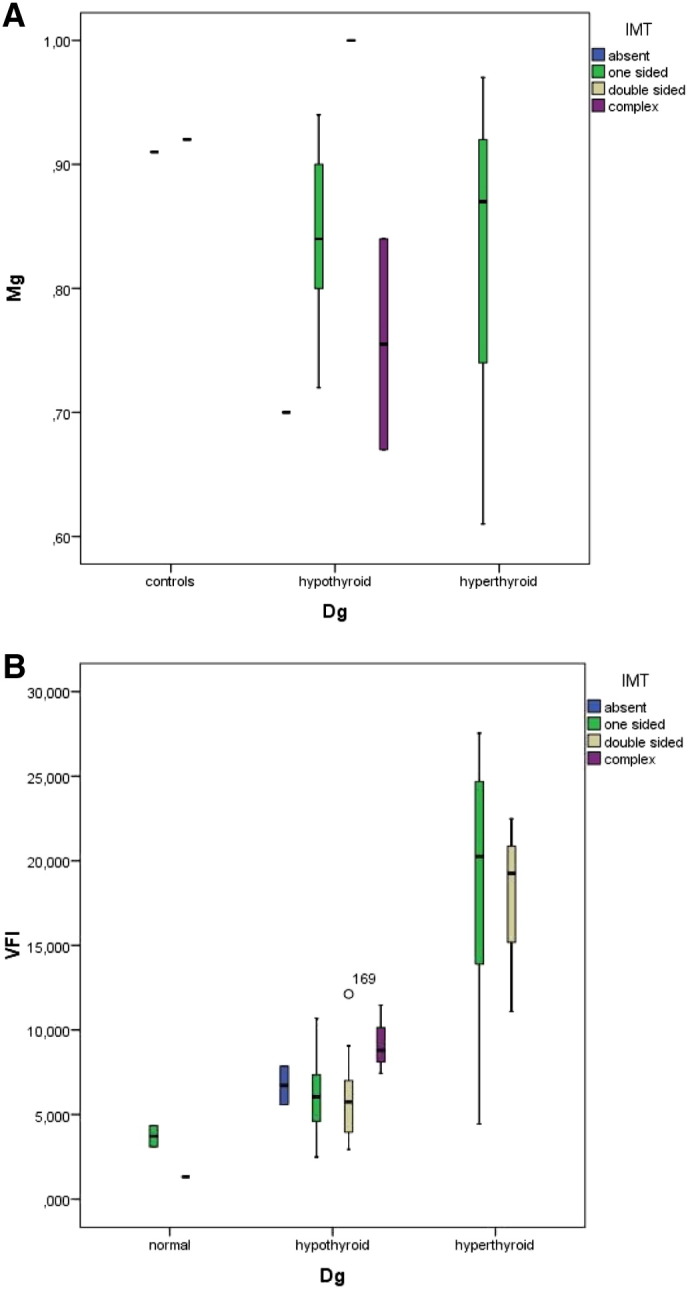
Reciprocal changes of magnesium levels and VFI in relation to findings of idiopathic moving toes.

In patients without evidence of lateral tension VFI was 5.93 ± 2.73, when single-sided lateral tension was found the VFI was 7.99 ± 6.91, and when lateral tension was bilateral the VFI was 11.44 ± 8.5. On the other hand the VFI levels showed no relation to IMT. The values found were: 10.62 ± 7.75 in one-sided IMT, 8.41 ± 5.7 in two-sided IMT, and 9.45 ± 2.86 in complex IMT situations. The opposite changes were seen between lateral tension and IMT in relation to serum magnesium levels (Figs. 6A–B, 7A–B).

Fig. 7 A and B show the interrelations between IMT condition to magnesium levels (A) and to VFI (B).

**Fig. 8:**
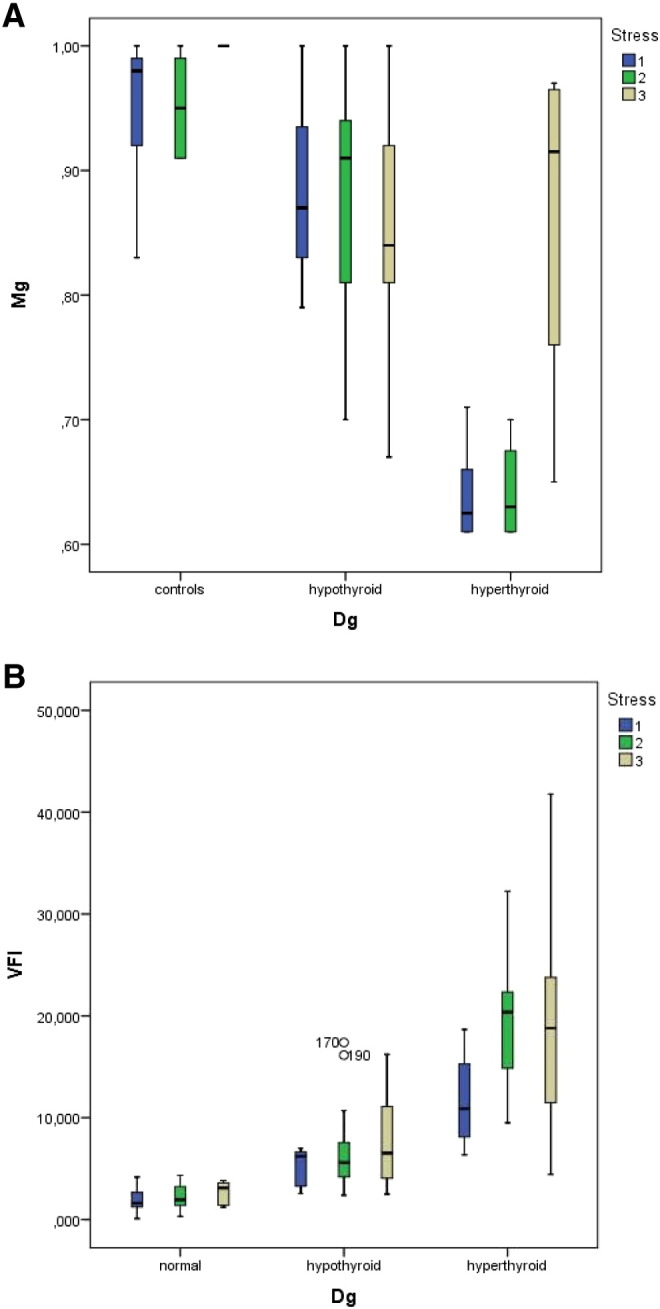
Reciprocal changes of magnesium levels and VFI in relation to findings of stress score.

We obtained similar results when plotting the values of the simple stress score and magnesium levels Fig. 8(A) as well as VFI, Fig. 8(B).

Magnesium supplementation led to a significant drop in the proportion of patients with IMT as this finding was no longer seen in 60% of cases after 6 weeks. Correction of the respiratory faults of the pelvis also diminished the amplitude of IMT movements. Lateral tension was not affected by magnesium supplementation. Lateral tension was very frequently seen in relation to anterior rotation of the hemi-pelvis of the affected side. For the following procedures the patients were lying supine on a treatment couch. These pelvic changes were corrected by manual therapy procedures using De Jearnette blocks as described elsewhere [22]. Treatment of lateral tension also required the use of acupuncture. This was done using neutral needling technique and 0.25 × 25 mm acupuncture needles (Hwato Master Touch, Suzhou Medical Appliance Factory, China provided by Bacopa, Austria). These individual acupoints called A-shi [40] correspond to hardened structures on the lateral side of the shank which was palpated during the foot rotation test. Anatomically they correspond to the soleus and lateral portions of the gastrocnemius muscles. Besides lateral tension and IMT, additional changes found frequently were a blockade of the normal pelvic motion during the inspiratory phase of respiration [41]. This blockade was treated with De Jearnette blocks in order to elevate the sacrum. During these therapy sessions the patients reported some tingling sensation on the shanks at the place where the acupuncture needles were placed. After completing the 20–30-minute treatment session the patients described a sensation of having heavy shanks, warmth in the pelvis, as well as tiredness. Finally a feeling of general lightness was reported when standing up.

### 3.8 Expanding the clinical picture via a-posteriori reconstruction

During the conduction of the study the patients were able to reconstruct data on their prior clinical history and symptoms. At the beginning the clinical symptoms included tachycardia, perspiration, muscle tiredness, nervousness, being easily fatigued, diminished physical potential, feeling unable to do sports due to unusual tiredness, muscle cramps, stiffness of the muscles, and postural instability. Further items included slight cognitive dysfunction being unable to concentrate, memory loss, and “jumpy” thoughts. Some subjects reported also heat sensation in the body and a sensibility towards a warm environment. The symptoms showed an improvement after correcting the musculoskeletal changes and taking the supplementation. The patients reported feeling fitter, having more energy, showing less fatigue, having a better sleep quality as well as correction of constipation. Single patients described less anxiety and less panic feelings. After the patients had been instructed about the nature of the musculoskeletal changes some of them were able to recall potential predisposing factors. The most frequent event could be identified as a situation of minor trauma in the form of grade I ankle sprain with supination–extension of the foot followed by a tendency to give-way. Such an event had occurred 5 to 8 years before, thus confirming our previous index case description [10]. These ankle sprain situations had required neither surgical care nor immobilization. Some patients also recalled having injuries of the sacrum while falling down backwards.

### 3.9 Sequential thyroid perfusion parameters in relation to hyperthyroidism, physical stress, psychological stress, and common flu

**Fig. 9:**
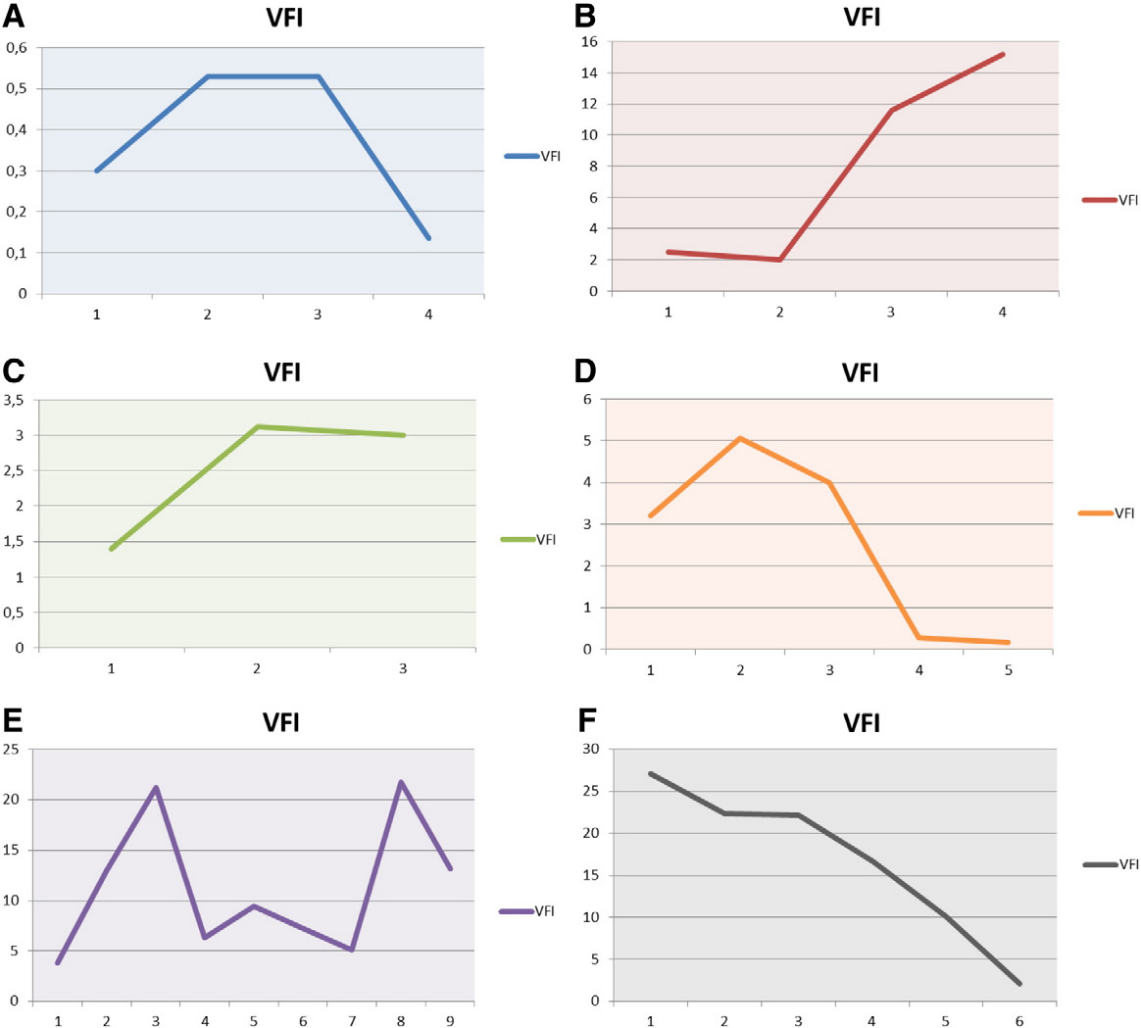
VFI values in relation to different stressors.

While the majority of patients were examined three times, single sequential cases were followed closer and more often (up to 8 follow-up examinations). This sequential follow up allowed us to recognize several conditions that showed an interaction with the VFI parameter. The six panels in Fig. 9 illustrate these interactions. The reader should keep in mind that the data presented in these images are plotted individually for each case and have thus different Y-axis values. Fig. 9A and B demonstrate the effect of physical stress in one trained and one untrained subject, respectively, who competed in triathlon. The first one showed a slight increase post-competition after an Olympic distance triathlon (1500 m swim, 40 K bike, 10 K run), however all values were always in the normal range. The second subject competed in a sprint triathlon (750 m swim, 20 K bike, 5 K run) without having sufficient previous physical training. The VFI increased significantly after the competition and remained elevated even 10 days post-competition. Fig. 9C shows a case of incipient hypothyroidism immediately following IVF and embryo transfer. The VFI increased to the upper range of normal and could be stabilized by means of selenium and magnesium supplementation. At the same time TSH levels returned to the normal range. Fig. 9D depicts the case of post-partum thyroiditis where the VFI was elevated. Following supplementation a clear improvement was seen together with normalization of thyroid function.

Fig. 9E depicts a period of 18 months of a patient who initially had hyperthyroidism. The initial VFI was only slightly elevated. During psychological stress due to a cumulative term examination (time point 3) during medical studies the VFI increased and could be normalized by supplementation. Later on a slight increase of the VFI was seen after a small trauma to the arm had occurred as consequence of a bicycle accident (time point 5). A second rebound was seen after the patient started taking an oral contraceptive containing ethinyloestradiol and chlormadinone (time point 8). After stopping the oral contraceptive the VFI diminished (time point 9). Fig. 9F depicts the course of a female patient with latent hypothyroidism that was treated solely with selenomethionine and magnesium supplementation. Under this protocol both VFI and TSH decreased.

**Fig. 10:**
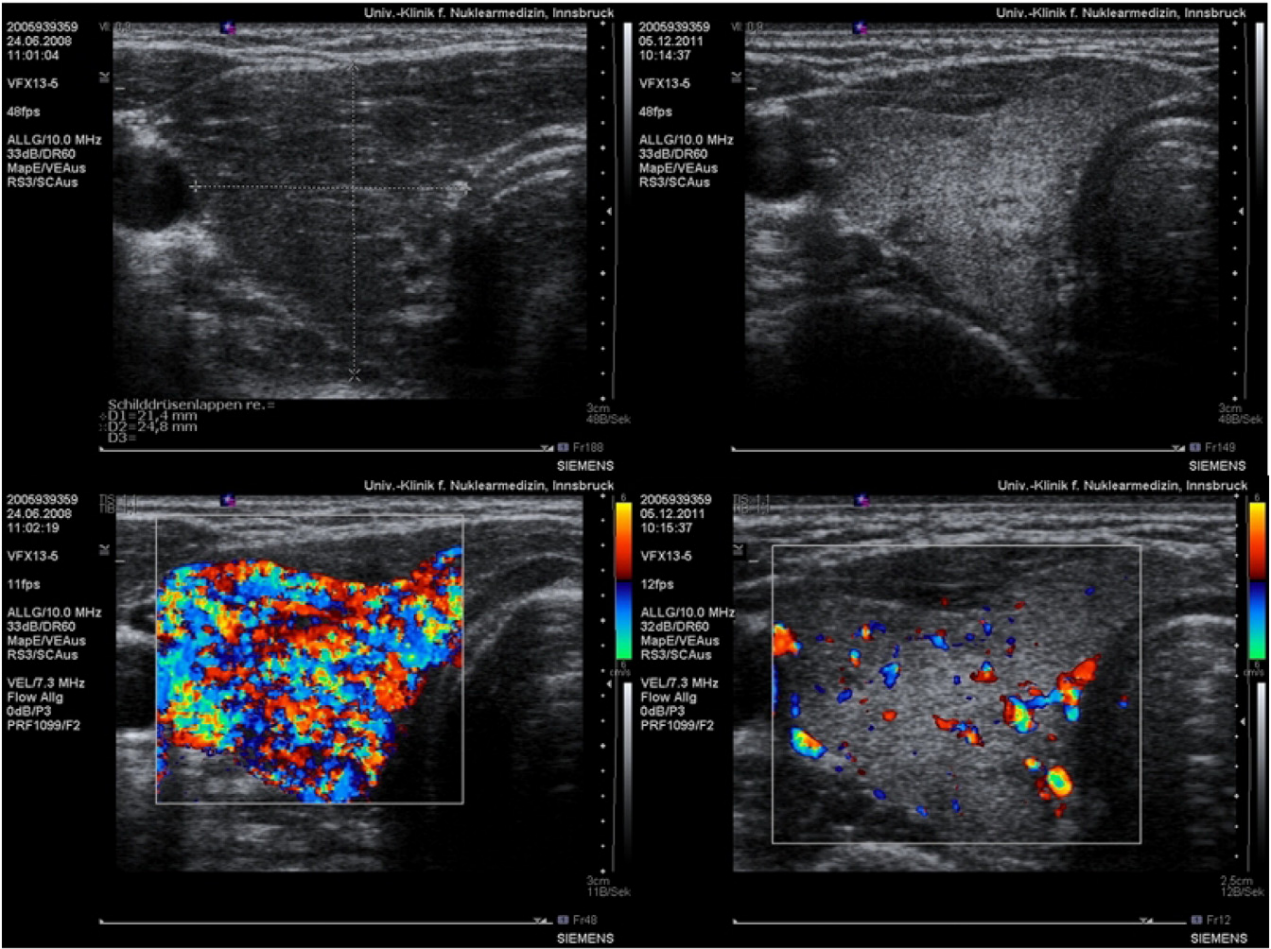
Improvement of thyroid morphology and vascularization following 3 years of supplementation with selenomethionine and magnesium citrate as well after correcting lateral tension and stress. Left pair initial situation, right pair follow-up after 3 years.

One case of a 26 year-old woman presenting Graves' disease was followed during 3 years. Under the supplementation regimen a steady improvement in thyroid function was observed. The most relevant change seen in this case was the normalization of thyroid morphology and perfusion as seen in ultrasound (Fig. 10). Parallel to these parenchymal changes the size of the thyroid diminished significantly. Surgery was no longer considered as an option.

**Fig. 11:**
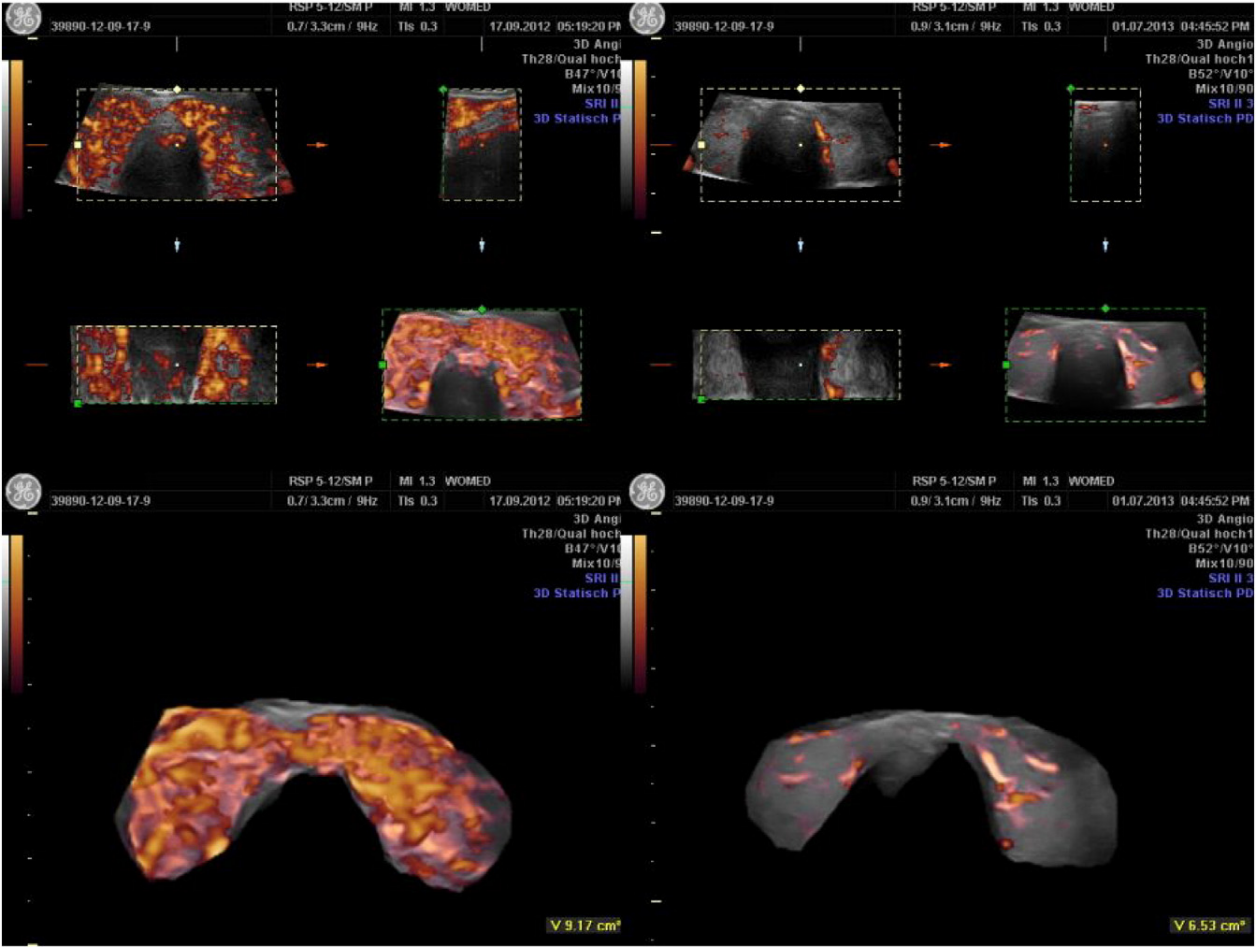
Demonstration of decreased vascularization in a female patient with hyperthyroidism following supplementation with magnesium citrate and selenomethionine over a period of 14 months. coenzyme Q10 (120 mg/d) was added in the last 4 months.

We had the unique opportunity to examine and treat three siblings, all females, of which 2 were twins. Initially one of the twins was diagnosed as having Graves' disease. The second twin looked for medical counseling in order to discard a similar affection, which was indeed present. The clinical and laboratory findings of the twins were extraordinarily similar during the whole course of treatment. Initially magnesium levels were 0.6 mmol/l. Supplementation was started with 8 tablets of a combined magnesium citrate plus glutamate preparation containing 60 mg elemental magnesium per tablet, i.e. This dose corresponded to 6.85 mg Mg/kg BW totaling 19.74 mmol/day. According to Seelig an adult requires between 5 and 10 mg magnesium per kilogram [42]. This strategy showed very modest clinical improvement. In view of this we prescribed a magistral formulation of pure magnesium citrate, 1.4 mmol/capsule t.i.d. Although magnesium levels did not increase under this dose thyroid hormone levels decreased by 20%. The third sibling was seen also in order to discard thyroid disease. At the time of presentation an ophthalmologic problem was present in the form of Leber hereditary optic neuropathy. Upon clinical examination we found similar postural changes as we have described in thyroid associated ophthalmopathy [8]. Her symptoms improved after correction of the postural changes together with a combined supplementation plan with magnesium citrate, selenomethionine and coenzyme Q10. In view of these results, coenzyme Q10 (120 mg/capsule, Pure Encapsulations) was also added to the supplementation of the twins. This triple approach brought a normalization of thyroid function together with an improvement in the morphology of thyroid vascularity (Fig. 11). It should be noted that the initial morphology of the vessels which appeared as thickened structures was no longer present at the end.

Finally one patient was studied during the beginning of a viral affection (common flu) which began short time after a routine medical check. Perfusion parameters were higher during the time of clinically evident flu.

### 3.10 Additional collateral clinical observations: liver function, tendinitis, learn stress and side effects of supplementation with magnesium citrate

**Fig. 12:**
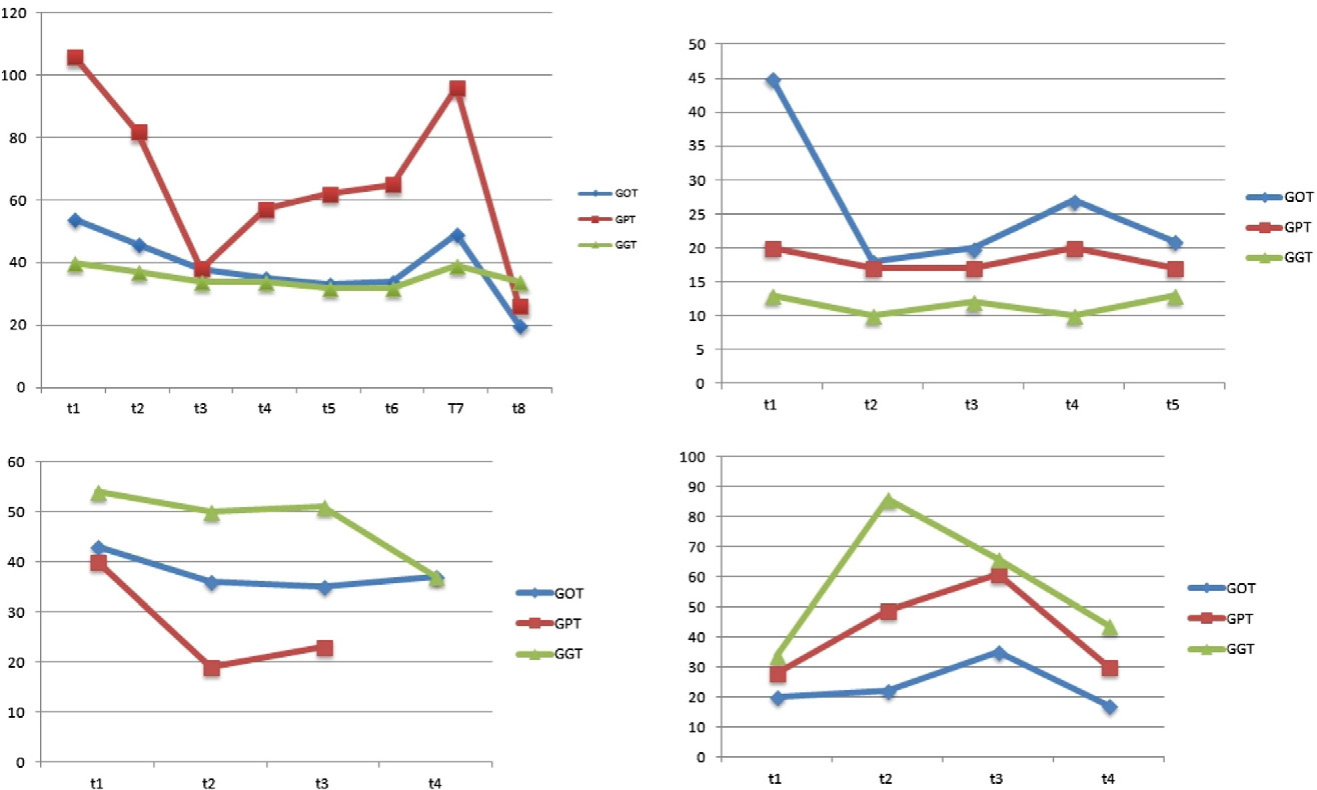
Beneficial action of the supplementation on liver function tests.

Since alteration in liver function parameters can be seen during initial phases of hyperthyroidism we followed-up 4 cases who presented initially elevated levels of transaminases. Under the same supplementation regime with magnesium citrate and selenomethionine there was a clear improvement of these parameters (Fig. 12). In two cases a rebound increase of transaminases was seen at a time when the patients stopped the supplementation.

A second important collateral finding was that of being able to improve tendinitis complaints by using a neutral cream formulation to which triiodothyronine (T3) was added. This empirical formulation was developed on the basis of clinical findings in 4 patients who had slightly elevated levels of fT3 in blood while presenting tendinitis. Based on reports on the anti-inflammatory effects of thyroid hormones on the skin [43], [44], [45] we designed a preparation containing 50 μg T3 in 25 ml of Ultrabas–Ultrasic. This approach was used in 29 patients presenting complaints of tendinitis of the flexor muscles of the arm as well as in 1 case of epicondylitis. Such a situation can arise from working on a computer and using a mouse on a relatively hyper-flexed position of the hand. Clinical improvement was seen in 29 cases within a period of 2 to 4 weeks of using the cream 3 or more times per day. The patient with epicondylitis showed no improvement and required local use of steroids.

A group of 8 euthyroid subjects presenting anxiety and panic condition due to learn-stress at high school or university because of upcoming important examinations were treated with 4 × 1.4 mmol magnesium citrate daily. They reported feeling calmer and been able to learn better within 10–14 days. This approach was conceived as a counter-measure to magnesium loss under such situations [46].

Concerning side effects we can evaluate now more than 2000 individual prescriptions of magnesium citrate over the last 2 years. We have observed two single cases of loose stools which disappeared after reducing the dose. Two subjects presented gastric hyperacidity.

## 4 Discussion

In this clinical study centered on patients with hyperthyroidism we have been able to find similar musculoskeletal changes and psychological stress factors as those recently described by us in patients with hypothyroidism [18]. Increasing levels of stressors are associated with decreased levels of magnesium in blood whereby patients with hyperthyroidism have the lowest levels as compared with cases of hypothyroidism. Together with this biochemical alteration we document increased levels of vascularity within the thyroid. Several stressors such as psychological and physical, e.g. high level of sports achievement in untrained subjects, as well as concurring infections had a negative influence on thyroid vascularity, i.e. perfusion parameters increased. On the therapeutic side we have demonstrated a beneficial effect of a combined supplementation regimen based on 200 μg of selenomethionine and magnesium citrate 3 × 1.4 mmol/d over a period of 3 months on thyroid function and on the intensity of thyroid vascularity. In view of these results, the null-hypothesis of our study has to be discarded.

An important issue for patients has also been addressed by our work: there was a clinical improvement in the well-being in the majority of cases after correcting the changes and deficits these patients presented. The patients reported feeling fitter, having more energy, showing less fatigue, constipation had disappeared, and they also reported a better sleep quality. Single patients described less anxiety and less panic attacks. We have continued to observe these positive effects in patients that now consult us directly because of remaining symptoms of thyroid disease in spite of being adequately treated, medically speaking. This leads us to conclude that the clinical symptoms in benign thyroid disease *are not* exclusively associated with thyroid hormone levels but rather with magnesium deficiency [18]. Magnesium supplementation showed a beneficial effect on thyroid economy leading to a normalization of TSH levels. These dualities in pathogenesis have not been described before in thyroid practice and add a new insight into thyroid physiology.

### 4.1 Implications that the biochemical mechanisms have for the observed clinical findings

A central clinical issue of our study relates to the definition of normal magnesium levels in blood. By having identified musculoskeletal changes and psychological stress as factors that lead to decreased magnesium levels, the reference range was built on the basis of healthy controls that lacked these stressors. We consider a reference range for magnesium of 0.7 to 1.0 mmol/l [47] as being inadequately wide and inaccurate because it includes very low levels. Unfortunately for medical research a considerable number of studies in the literature have worked with such reference levels. In our opinion the reason for this large discrepancy is that the definition of reference values for clinical chemical parameters did not include a detailed physical examination of the subjects. Large number of anonymously obtained laboratory values cannot replace a clinical evaluation. In addition to this clinicians have to be aware that magnesium intake has decreased in recent time [48] making hypomagnesaemia maybe more probable and frequent. Finally studies that have evaluated magnesium levels in different settings might have to be re-appraised. Using a theoretical approach Liebscher and Liebscher have provided an example of re-appraisal. They re-analyzed available data on magnesium levels and concluded that adequate reference values should have a lower limit of 0.9 mmol/l [49] which is close to our calculated mean reference value. On the therapeutical side we differ from their point of view regarding the dose needed for substitution given as 600 mg of magnesium daily. Unfortunately this publication contains no details as to the exact formulation of the magnesium salt that should be used. Additional discussion of this aspect is included in Section 4.4.1.

### 4.2 Correlation between magnesium and selenium and hepatic function

It is interesting to remark that a positive correlation between whole blood selenium and serum magnesium was lost in situations of thyroid disease, sterility, depression and infection. This situation suggests that blood magnesium levels seem to be related to an adequate selenium uptake. An indirect hint can be found in the description of magnesium saving action of the selenoprotein MsrB1 acting on TRPM6 [50]. On the other hand, our preliminary data on improvement of hepatic function parameters after supplementation coincides with the experimental results reported by Markiewicz-Górka et al. [51]. The improvement in liver function observed after supplementation in a small group of patients is a novel finding in the field of hepatology. In 1975 McConnell et al. described a lower Se content expressed in μg Se per gram dry weight in patients with “diseased liver” [52]. In 1979 Sullivan et al. described the concomitant finding of low levels of Mg and Se in patients with cirrhosis [53]. Other authors also described decreased levels of Se in alcoholic cirrhosis [54], [55]. In an experimental model presented by Li [56] an increased content of lipid peroxide and hydroperoxide were found in guinea pigs with selenium deficiency. In 1993 Rayssiguier et al. [57] described the influence of magnesium deficiency to an increased level of peroxidation of lipoproteins. In 2001 Loguercio et al. [58] found decreased levels of Se in relation with chronic liver damage. Navarro Alarcón et al. [59] described a negative correlation between Se and liver function parameters such as glutamic–oxalacetic-transaminase and gamma-glutamic-transferase. In the field of oncological epidemiology Strasak et al. [60] described a relation between elevated gamma-glutamic-transferase and cancer incidence in women. These studies, however, have not developed any therapeutic recommendations.

### 4.3 Magnesium levels and ATP synthesis

Based on medical literature, we have previously hypothesized that low magnesium levels could affect both ATP synthesis [61] as well as telomerase activity [18]. The interaction of low levels of magnesium with telomerase activity has been demonstrated recently in-vitro by The Alturas and collaborators [62]. Our supplementation procedure with magnesium citrate and selenomethionine thus gains importance in a wider context of clinical situations. In the first place it could contribute to an improvement in the treatment of mitochondrial diseases, where magnesium is often not even recalled in schematic representations of the function of Complex V of OXPHOS [63]. Clear-cut descriptions of the importance of magnesium in relation to ATP can be found in other recent publications [64], [65], [66], [67]. Vinogradov has described the regulation of F_1_F_O_ATPase and the role of magnesium [19]. The reaction of mitochondrial H + -ATP synthase reads:

$$\overset{\mathrm{Mg}}{ADP+\mathrm{Pi}+nH^{+}\mathrm{out}+mH+\to ATP+H_{2}O+nH^{+}\mathrm{in}.}$$

Further literature describes the interaction of magnesium on F1F0ATPase [68] and the relation of magnesium to the nucleotide binding sites of F1F0ATPase [69].

### 4.4 How new are our findings? Historical review on magnesium and thyroid disease

The oldest report we have found that deals with the use of magnesium for treating hyperthyroidism was published by Hueber in 1939 [70]. The author based his approach on experimental observations relative to a down-regulation of cellular respiration of the cardiac muscle of the frog as well as to a slowing down of energetic processes. Hueber treated 6 patients who presented tachycardia, increased perspiration, weight loss, tendency towards being easily aroused, and tremor. All of them had an elevated basal metabolic rate. In 2 cases the hyper metabolic condition developed after an infection. The treatment consisted in a t.i.d. administration of 1.5 g of magnesium glutaminate, either i.m. or i.v., over three days. After this treatment the initial high values of basal metabolic rate fell from 125–160% to 107–113%. The patients reported a feeling of well-being and cardiac symptoms disappeared. Only one female patient required a longer treatment. Due to persistence of hyper-metabolism she was submitted to thyroid surgery. The histology revealed a toxic adenoma. The results were explained in the light of the work of Warburg on quantization of cell respiration [71], [72].

In 1961 Wiswell studied the effects of 1.0 g magnesium sulfate, an inorganic salt given b.i.d. to 2 patients with hyperthyroidism without seeing beneficial effects. One patient with hypothyroidism was treated first with 1.0 mg of triiodothyronine and then magnesium sulfate. The author observed a marked rise in urinary excretion of magnesium. In 1963 Neguib reported the use of magnesium sulfate as well as of magnesium chloride [73]. The dose given to 2 of his patients was 20 ml of a 25% solution by deep intramuscular injection. The patients reported that the injections were painful. The following patients were treated successfully with magnesium chloride beginning with a 1% solution. The dose was later increased to a 14% solution. The patients treated this way showed an improvement of their symptoms while at the same time goiter size was reduced.

#### 4.4.1 How to choose an adequate magnesium salt for supplementation

We would like to stress the implicit differences of the pharmacological preparations: good effects were observed by Hueber when using a glutaminate, whereas Wiswell found no changes while working with an anorganic salt, i.e. sulfate. This leads us to the question whether all these approaches can be compared? While the use of supplementation with magnesium to treat musculoskeletal changes has been rarely described, several studies have looked at metabolic derangements. Most published studies have used non-organic magnesium salts such as sulfate or oxide. In 1982 Dyckner and Wester gave a general recommendation as to administer 30 mmol of magnesium sulfate intravenously over 12 h [74]. Oster and Epstein recommended magnesium oxide up to 4 times 12.34 mmol [75]. Using magnesium pidolate, Paolisso et al. administered 16.2 mmol to adult patients aiming at an improvement of glucose handling [76]. Lima et al. administered either 20.7 or 41.7 mmol of magnesium oxide to patients with type 2 diabetes [77]. Ayuk and Gittoes advocate the i.v. use of magnesium sulfate starting with doses of 8 to 12 g (corresponds to 32.91 to 49.47 mmol magnesium) [78]. It is clear that the doses of anorganic magnesium salts are exceedingly higher than those of magnesium citrate. The reader should be aware of the beneficial effects of magnesium citrate on energetics and the importance of the citrate transporter for mitochondrial homeostasis [36], [37], [79]. This appears to us to be of central importance in our study since hypothyroidism affects negatively the mitochondrial citrate carrier activity [80].

The importance of citrate has been described by Icard, Poulain, and Lincet recently [81]. In their publication they stress the functional importance of citrate in mitochondrial function and also mention that citrate “also acts as an “energy gauge”, a powerful sensor and regulator of cell energy production, adjusting both production and need” (page 115 in [81]). Citrate is an important carrier of acetyl groups from the mitochondria to the cytoplasm [82]. The human sodium-coupled citrate transporter has been described as an important player in the generation of metabolic energy [83].

### 4.5 Magnesium and thyroid function

Besides these data on the therapeutic use of magnesium, information on a relation between magnesium and thyroid function can be found in publications which appeared some 60 years ago. Experiments that aimed at interfering oxidative phosphorylation in thyroid samples were conducted in 1955 by Freinkel and Ingbar [84], [85]. Using disruptors such as 2,4-dinitrophenol or cyanide the iodide concentrating capacity of the thyroid was lowered. In 1968 Tyler et al. investigated the effect of mitochondrial inhibitors on the energy-dependent uptake of iodide by thyroid slices [86]. The inhibitors oligomycin, antimycin, and rotenone lowered the tissue to medium ratio of iodine, which reflects iodine uptake, and inhibited also oxygen uptake. The tissue to medium ratio fell from 38.1–45.5 in the controls to 2.8–3.2 for oligomycin, 1.6–2.2 for antimycin, and to 1.5 for rotenone. Using nucleotide determinations they found a significant drop of nucleoside triphosphate content in thyroid slices when the inhibitors were used. They concluded that endogenous ATP synthesis appeared to be essential for iodide uptake to take place. While glycolytic ATP synthesis was able to provide energy for iodide uptake, exogenous ATP had no effect. It should be kept in mind that oligomycin is an inhibitor of Complex V of the respiratory chain where ATP synthesis takes place [87]. In 1972 Heaton and Humphray [88] observed under experimental conditions that the uptake of radioactive iodine by thyroid glands depends on magnesium since magnesium loading stimulated this process while magnesium deficiency resulted in an inhibition of uptake. These effects would explain the changes in TSH levels which we found after magnesium supplementation. Surprisingly we observed no constant pattern of change for fT4. Both hormones appear to have a complex interrelationship [89], [90], [91]. The role of magnesium in this context has not been investigated.

### 4.6 Thyroid function and oxidative phosphorylation

We would like to describe other studies that dealt with the relation between thyroid function and oxidative phosphorylation and which appeared in the time period 1951 to 1957. Some key findings were the uncoupling of respiration and phosphorylation by thyroid hormones [92], [93] which was accompanied by swelling of the mitochondria [94] while magnesium administration displayed an antagonistic effect [95]. In 1951 Carl Martius and Benno Hess measured phosphate esterification and demonstrated an inhibitory action for thyroxine [96]. This effect resulted from the use of a pharmacological dose of the hormone, i.e. 6 mg, for rats weighing approximately 120 g. This corresponds to 50 μg per gram. In 1954 Hoch and Lipmann published a study entitled “The uncoupling of respiration and phosphorylation by thyroid hormones” [92]. The authors utilized tissues from animals that were artificially made hyperthyroxinaemic. The animals used were young male Syrian hamsters having a mean weight of 100 g. The dose used was of 4 mg in 0.5 ml on the first day followed by 8 mg in the following 3 day making a total dose of 28 mg in 4 days. This total dose corresponded to 280 μg per gram weight. It should be noted that in human medicine the usual thyroxine substitution dose lies between 100–125 μg for a subject weighing approximately 70 kg.

In 1962 two further papers appeared [97], [98]. Hoch here discussed the nature of thyrotoxicosis as being a disease of mitochondria. He recognized that the doses of thyroxine used in experimental settings are quite high (page 447 in [98]). Referring to “… the paradoxical metabolic and physical inefficiency of thyrotoxic subjects… suggests that thyrotoxicosis is a disease of defective energy transfer” (page 448 in [97]). In the discussion he describes the uncoupling of oxidation and phosphorylation which results in inefficient energy utilization. An explanation was not given in the publication. In the second paper, he discusses the fact that hyperthyroidism is associated with glycogen depletion [97]. He also discusses the biochemistry of skeletal muscle which depends upon oxidative re-synthesis of ATP for regeneration of ATP. In a short discussion Hoch touches the topic of magnesium in relation to OXPHOS and concludes that this area of investigation needs to be clarified.

Vitale et al. reported that thyroxine administration had an effect on magnesium requirements, i.e. an increased demand [99], and that the changes seen after administering thyroxine were similar to those seen in conditions of magnesium deficiency [100]. Again the dose of thyroxine used in these experiments was quite high, i.e. 1 to 4 mg per 100 g diet. In those experiments where magnesium was administered, one cannot exactly reconstruct how much elemental magnesium was actually given. OXPHOS was impaired in the thyroxine treated animals and this change was repaired by magnesium supplementation. Weight gain of the rats was maximal with 80 mg % magnesium and lowest when 4 mg % of thyroxine was given. Both magnesium deficiency as well as thyroxine excess produced signs of magnesium deficiency in the sense of vasodilatation and hyperemia [99]. In the experiments with magnesium deficiency, cardiac muscle was more sensitive than kidney and liver showing a decrease in OXPHOS. The injection of thyroxine also produced a fall in OXPHOS and this change could be prevented by administering magnesium [100]. In view of these results Vitale et al. proposed that hyperthyroidism leads to an increased magnesium requirement.

In 1962 Corradino et al. [101] summarized some of these early observations and concluded that magnesium may serve to regulate the thyroid function itself. In their own experimental work they found an increase in the size of the thyroid under conditions of low magnesium. In 1966 Jones et al. presented a review on the topic of magnesium levels in relation to thyroid function [102]. In hyperthyroidism urinary excretion was elevated, and the opposite was found in cases of hypothyroidism. Treatment of the corresponding thyroid diseases normalized the excretion values. It is interesting to recall that they observed a higher excretion rate of magnesium being achieved by T3, which is similar to the effect recently described by Rodrigues Dare [103]. In 1968 Hoch stated the reciprocal relation between thyroid hormones and magnesium as follows: “Magnesium metabolism depends upon the thyroid state, and vice versa. Mg^++^ and thyroid hormones are antagonists in vivo and in vitro when mitochondrial function is measured” (page 357 in [104]). In 1996 Disashi et al. described a negative correlation between fT3 and Mg [105]. These details and our findings indicate that current concepts on thyroid hormone action might have to be re-evaluated [106].

### 4.7 Development and expansion of the WOMED model: the pathway to an acquired alteration of mitochondrial function affecting Complex V of oxidative phosphorylation

#### 4.7.1 Ankle sprain grade I as the trigger

We postulate that the chain of events involved in the development of thyroid disease begins with an ankle sprain grade I lesion. Since this slight lesion does not require medical intervention not all patients can immediately recall such an event, thus this information can be temporarily lost. Having a grade I ankle sprain body weight is shifted to the unaffected body side. We have called this situation lateral tension [10]. We have been the first authors to describe these changes and have a complete follow-up of the index patient. The affected limb assumes an eccentric muscle position with an outward rotation of the leg and an anteriorly rotated iliac spine. This lateral tension affects the shank muscles, i.e. the triceps surae and the soleus, a muscle group that has a special meaning in body support [107], [108] thus being challenged every day in its anti-gravity function. Both the soleus and gastrocnemius muscles are involved in producing ballistic bias impulses in relation to human postural sway [109]. The amount of muscle activity during the day can vary according to its intensity [110]. This challenge is not of great daily magnitude per se, however the muscular consequences add up through time. We have seen that the time at which ankle supination lesions occurred was longer than 5–10 years. Our evaluation of body weight distribution shows a slight displacement of 2–3% of body weight away from the affected side. For a person with 70 kg body weight this would correspond to adding 1.4 to 2.1 kg of weight to the unaffected side. The ensuing bioenergetics of such a body shift has have been described elsewhere by us [18]. Adding a similar amount of weight to a limb will result in an increased metabolic rate [111], [112]. In short form there is higher energy expenditure and the main resulting symptom can be fatigue which becomes one of the main complaints of the patients.

Two interesting aspects of ankle sprain have been described in a recent publication made by the International Ankle Consortium [113]. The first one refers to the lack of medical care in the majority of cases; the second one describes the long term sequelae calling them a large healthcare burden. We firmly believe that our approach could help to improve outcome. We have found no studies that looked at thyroid function following ankle sprain [114], [115], [116].

In addition to lateral tension the coordinated movement of the diaphragm and pelvis [117] can be affected due to a blocked pelvic movement in the inspiration phase due to a backward tilted pelvis. Although we have not found any biomechanical investigation on this topic it is logical to consider this backward tilt as a condition that increases the physiological angle between the pelvis and the legs, i.e. stretching, and might be contributing factor for the development of IMT. Both lateral tension and IMT are associated with low levels of magnesium. IMT can be corrected by magnesium supplementation alone in a great number of patients. Treatment of lateral tension requires manual medicine procedures and acupuncture.

Besides these biomechanical elements one also has to consider local changes after the supination lesion. The initial lesion induces a flexion–supination stretching of the shank muscles putting strain on the bony insertions sites resulting in muscle tear. This tear lesion is associated with micro-hemorrhages that can be found after using acupuncture at the site of the lesion [118]. The constant eccentric muscle action during every day activities could add through the years more shear stress to the shank. This event can be viewed to be similar to crush muscle injuries where pressure- stretch changes occur. Ionic fluxes activate energy-dependent mechanisms — which will require inevitably magnesium. A depletion of ATP will follow [119]. Local perfusion is increased in such lesions [120] by which signal molecules will be transported systemically.

This local event activates the danger elements in front of an anatomical lesion and will also have as a consequence a stimulation of the immune system as can be demonstrated under experimental conditions [121] or in the context of the “danger theory” put forward by Polly Matzinger. One key concept in this theory is that cellular alarm signals arise endogenously from distressed or injured cells [122], [123]. This species of molecules are called damage associated molecular pattern (DAMP) molecules which undergo denaturation once they are released from the affected cell [124]. According to our model, we would expect that the local influence of injury will be then followed by mitochondrial distress. Mitochondria in distress could turn to be sources of inflammatory signals [125]. Additional signals that could be released from the lesion include myoglobin. Myoglobin levels have been described as being altered in cases of thyroid disease [126], [127]. Myoglobin has been detected also in thyroid tissue [128], [129]. Patients with thyroid diseases can have anti-myoglobin antibodies in blood [128], [130].

#### 4.7.2 Anxiety, cognition, telomerase activity and the role of psychological stress

A further central issue involved in this complex is psychological stress which needs to be identified and treated. While we focus our discussion on thyroid disease recent data has pointed out the effect of adversities in early life which influence physiological regulation in later life [131]. While the original theories of Selye on the general adaptation syndrome as a response to a general alarm reaction [132] initiated research on the hormones of the HPA, recent experimental research has clearly demonstrated the endocrine response pattern associated with stress reactions can be triggered by a condition of magnesium deficiency. This leads to a situation of anxiety and to activation of the HPA axis [133]. Altogether we believe that is sensible to propose that as a consequence of the negative effects of the physical and psychological stressors magnesium levels decrease creating a situation which increases the general susceptibility to stress [134]. On the other hand and contrasting with chronic stressors, acute stress has been considered to be beneficial as it stimulates neurogenesis in the hippocampus under experimental conditions [135].

Cognitive impairments and fear generalization have some common elements that are linked to magnesium, selenium and coenzyme Q10 as well as to the hippocampus. In order to describe these links we cite here some clinical and experimental data in a chronological order which corresponds roughly to life development. The main terms describing this setting are maternal prenatal stress and early life stress. In a similar way research on epigenetics goes even to consider a broader time window. Citing Lo and Zhou [136]: “There are three categorical stages of life history when epigenetics are registered — ancestral (including parents), prenatal, and postnatal stages. Among these stages, prenatal epigenetic registration is the most eminent and profound influence on the formation or fine-tuning of the nervous system during development.” We consider that the starting point in clinical work should consider prenatal maternal stress which can be seen as a contributing factor to stress conduct later in life [137], [138], [139]. These effects show changes in the function of the hippocampus [140]. Early life stress has been found to shape areas of the brain related to emotion processing located on the hippocampus and amygdala [141] and early life stress can be related to hippocampal cell loss [142] as well as to inhibition of neurogenesis [143]. The timing of these situations, i.e. pregnancy [144], is directly related to the concept of pre-natal stress. Ante-natal stress has also been considered as a possible origin of later psychopathology [28], [145]. Both intellectual functioning and cognitive ability or development can be affected in children [146], [147], [148], [149], [150]. Contrary to pre-natal stress, maternal care promotes hippocampal synaptogenesis and in the end cognitive development [151]. This function is related to the brain-derived neurotrophic factor (BDNF). Selenium exerts neuroprotective action on the hippocampus in relation to BDNF [152]. One important mechanism that is found in cases of maltreatment is DNA methylation of BDNF [153]. Szyf considers DNA methylation a mechanism by which experiences in early life can be embedded [154], [155]. Experimental magnesium deficiency during pregnancy has been found to be related to hyper-methylation [156]. An additional mechanism related to intrauterine stress is the appearance of shorter telomeres in young adults [157]. The Alturas have recently demonstrated that short-term magnesium deficiency can down regulate telomerase [62]. In addition to this, Bachnas et al. have shown that antenatal supplementation with magnesium sulfate in humans can improve the levels of BDNF [158]. In the setting of experimental menopause, Sandhir et al. have found that coenzyme Q10 can ameliorate cognitive functions [159]. Low selenium levels in elderly people can be associated with cognitive decline [160]. Exciting experimental data have been recently presented by Abumaria et al. on the beneficial effect of magnesium supplementation using a new compound magnesium l-threonate. It is effective in preventing fear over-generalization [161] and it can also enhance the retention of the extinction of fear memory [162]. Wang et al. have observed that magnesium l-threonate can prevent and restore memory deficits related to neuropathic pain involving TNF-alpha [163]. Cognitive functions such as learning can also be positively modulated by magnesium administration. This last action is related (again) to changes in the hippocampus [164]. These experimental findings are of interest to our research because they correlate with the effects of thyroid function on the hippocampus [165], [166], [167], [168] as well as to psychological stress due to trauma [46], [134], [169], [170]. As a corollary of these data we propose that magnesium deficiency could be involved in altered cognition development, an issue that has been seen almost exclusively to be related to thyroid function. An additional aspect to be kept in mind is the pattern of brain metabolism: synaptic growth gene expression is related to aerobic glycolysis while synaptic transmission is related to oxidative glycolysis [171]. Age differences in the metabolic patterns in the brain are apparent [172], thus injuries occurring at different ages might have other sequels.

#### 4.7.3 Magnesium, energetics and the immune system

Our observation of altered thyroid perfusion in relation to concurring infection made us curious about the energetic aspects of the immune system. The fine tuning of T-cell function and energetics has been summarized by Pearce et al. recently [173]. Magnesium even assumes the role of a second messenger that couples receptor activation [174] and is also involved in cytotoxic functions of NK cells [175]. Proliferative transition in lymphocytes are regulated by TRPM7 [176]. In 1994 Meldrun, Ayala and Chaudry described that administering ATP-MgCl_2_ led to improved lymphocyte energetics in late sepsis [177]. Both TRPM6 and TRPM7 are closely related to magnesium metabolism [178], [179]. On the side of pathogens, their spreading is also an energy consuming process [180]. According to Straub the chronicity in an infectious situation will occur when energetic reserves of the organism have been consumed [181], [182]. One has to view this consumption as part of the energetic demands which are needed for the immune response. In view of these modern data, it is not surprising that hyperthyroidism could develop following an infection as was described by Hueber [70]. These processes could explain the generally known symptom of malaise that is associated with viral infections. It follows that adequate magnesium levels are necessary for the maintenance of a well-functioning immune system.

#### 4.7.4 Mitochondrial function and physical fitness

Some modern studies have also investigated the relation between hypothyroidism and mitochondrial function. Siciliano et al. found an altered mitochondrial respiratory function in patients with hypothyroidism [183]. Although the authors described the presence of musculoskeletal symptoms as being related to the legs they examined biopsy samples taken from the left deltoid muscle. Analysis of muscle samples revealed a decrease in cytochrome c oxidase activity as well as a reduction in the levels of mitochondrial transcription factor A. In a later study they described the lack of effect of treatment with levothyroxine on muscle function in patients with hypothyroidism. Their study relied on parameters such as glucose tolerance, insulin response to glucose, resting heart rate, resting and maximal VO_2_, and maximal power output [184]. Contrary to the philosophy of our WOMED model approach, musculoskeletal symptoms were collected by a questionnaire, i.e. by means of hands-never-on the patient. We feel that a simple manual examination is imperative in this type of studies. A similar situation of hands-never-on the patient can be found in the field of sports medicine. In one study elite athletes attempted to get a satisfactory answer for their tiredness and recorded their answers on questionnaires [185]. A high level international consensus report on how to approach the over-training syndrome discarded a-priori the importance of magnesium levels [186]. It is known, however, that strenuous exercise will exert a negative influence on magnesium levels [187].

#### 4.7.5 Muscular damage and muscle repair: the role of thyroid hormones

What puts skeletal muscles and thyroid together? In relation to thyroid function, one has to differentiate two conditions: hypo- and hyperthyroidism. Low levels of magnesium could impair the capacity of the thyroid to import iodine actively thus leading to hypothyroidism. On the other hand, patients with hyperthyroidism present more musculoskeletal alterations due to altered axial orientation of the body [8], [9] which will consequently demand more repair mechanisms. Muscle repair belongs to the scope of action of thyroid hormone [188], [189], [190], [191]. In addition selenium deficiency can affect the concentration of this substance in muscular tissue [192]. We propose that the load of muscle mass needing repair will put a higher physiological demand on the level of thyroid hormone production [193], [194], [195] leading to a hyperthyroid state. This demand has to be met in an environment of selenium deficiency [11] and knowing that selenoproteins are important for skeletal muscle [196], [197] muscle repair might also suffer from selenium deficiency. Due to selenium deficiency the action of deiodinases can be low at the tissue level thus “forcing” the thyroid to produce a hyperthyroid state that produces preferentially triiodothyronine. Experimental data show clearly the effects of T3 in the maturation of muscle satellite cells [195]. In this system the differentiating muscle satellite cells produce FOXO3 which then stimulates the synthesis of deiodinase II [193]. Levels of deiodinase II are higher in slow twitch muscle cells [198]. Looking at the depiction of this process in a recent review [195] it is fundamental to add the fact that in turn FOXO3 is activated by AMP kinase (AMPK) [199]. AMPK functions as a metabolic sensor responding to a drop in ATP levels [200]. Here we add the notion that ATP levels diminish when magnesium levels are low. In addition to this, selenium intervenes again in this system adding a stimulatory action by activating AMPK as seen in experimental data from colon Ca cells [201]. In order to have physiological effects of selenium, it is important that hepatocytes maintain a good functional level in order to produce selenoprotein P [202]. Sepp1 needs to be in the long form, i.e. containing more than 6 Se-cysteine moieties, in order to be taken into the cells via the ApoER2 receptor [203], [204]. As we have shown in a small group of patients, supplementation with selenomethionine and magnesium can lead to improved hepatic function thus potentially influencing selenoprotein P synthesis. Another interesting fact is that FOXO3 is also involved in the regulation of mitochondrial function [205], thus bringing us back to the site of oxidative phosphorylation. De Lorgeril and Salen have discussed the importance of a functioning deiodinase system in order to achieve myocardial repair [206]. This action should also apply to skeletal muscle. Magnesium deficiency can also affect myogenesis [207].

One important issue to keep on mind is the need of mitochondrial biogenesis with increased citrate synthase activity for muscle regeneration to occur [208] (please refer to Section 4.4.1 on the choice of an adequate magnesium salt for supplementation). In turn thyroid hormones are effective on mitochondrial biogenesis as well as on muscle repair [209], [210], [211].

#### 4.7.6 Hypothesis: thyroid disease as an acquired dysfunction of mitochondrial function

Contrasting with genetically determined mitochondrial disease we propose that thyroid disease is a mitochondrial dysfunction entity related to an acquired condition of magnesium deficiency. Low levels of magnesium will produce an uncoupling of oxidative phosphorylation which is potentiated in cases of hyperthyroidism. All of these mechanisms compromise the function of the respiratory complex V leading to a decrease of the energetic capacity of the body.

Additional negative contributions can be due to low levels of coenzyme Q10 which in turn can be the result of low selenium levels [212], [213]. An additional factor which we have observed is increased vascularity under oral contraceptives. These pharmaceuticals have been found to influence negatively coenzyme Q10 levels [214], [215], [216]. Low selenium levels can also compromise mitochondrial biogenesis [217], [218]. In turn the selenoprotein thioredoxin reductase is an essential element that reduces coenzyme Q10 [219], [220]. In a neuronal model of coenzyme Q10 deficiency a reversal of ATP synthase activity was observed [221]. On the therapeutical side coenzyme Q10 can influence positively mitochondrial function [222], as well as angiogenesis and endothelium function [223], [224], [225]. This action on angiogenesis is said to be due to an interaction with fibroblast growth factor. Independent studies done with thyroid tissue have indeed shown this combination of findings: low levels of coenzyme Q10 [226] and elevated levels of FGF [227]. Our studies can be seen as a demonstration of the role of magnesium, selenium and coenzyme Q10 in vascularity. Recently, Kharitonova et al. have shown a beneficial effect of magnesium supplementation in restoring endothelial function [228]. It follows that the combined use of coenzyme Q10 and magnesium can be seen as an instrument to approach vascular disease. On the other hand, the hypothesis that thyroid hormones alone regulate vascularity, as discussed by some authors [229], has to be taken with caution since the data come frequently from in-vitro models that have used very high doses of thyroxine. One early work on myocardial hypertrophy following thyroxine administration utilized a dose of 250 μg/kg body weight s.c. per day. According to data from the RGD and data from Kurtz and Morris [230], the mean weight at 13 weeks would be 242 and 174 g for male and female rats, respectively. Using these figures the daily dose of thyroxine would be 60 μg and corresponds almost to the daily dose an adult person would need (dose app. 1 μg/kg body weight). Recently Freitas et al. have reported changes in the myocardium utilizing a high dose of thyroxine (600 μg/kg/day which corresponds to 150 μg/d per animal having a weight of 250 g). An extrapolation of these results to any human condition is not conceivable on physiological terms [231]. The same applies to the studies on thyroid hormones and oxidative phosphorylation (Section 4.6).

#### 4.7.7 Shortcomings found in studies on mitochondrial disease

A systematic shortcoming on the description of oxidative phosphorylation is the avoidance of magnesium in the description of OXPHOS. Few publications can be found that deal explicitly with Complex V [232]. Quoting De Los Rios Castillo: “The oxidative system (respiratory chain) couples redox reactions to the production of a proton electrochemical gradient, which drives the synthesis of ATP by the phosphorylation system (F_O_F_1_-ATP synthase and the ADP and phosphate carriers)”. It has been suggested that the presence of cristae in mitochondria depends on the ability of complex V to form dimers [233]. On the other hand, lack of magnesium will result in mitochondrial swelling. Magnesium deficiency is also related to an altered cellular composition such as a decreased number of mitochondria. Magnesium restores mitochondrial membrane potential [234]. Beard has described a biophysical model of the respiratory system of the mitochondria. On page 0255 he describes the fluxes involved in magnesium binding (Eq. 12 in [235]). Few studies have documented changes in mitochondria in relation to thyroid disease. Hypothyroid myopathy has been found to show absence of mitochondria [236]. A case of myopathy in relation with selenium deficiency was described as being associated with muscle weakness [237]. Recently interest on mitochondrial function has been described in the field of infertility [238]. Bloom et al. have shown that fecundity is related to magnesium levels [239]. This topic remains to be investigated.

#### 4.7.8 A short corollary on thyroid disease and magnesium

Elaborating on the experimental data mentioned in the discussion we propose the hypothesis that thyroid function is related to magnesium availability. Lack of magnesium will affect iodine transport which depends on energy delivered by magnesium-ATP resulting in hypothyroidism. Due to the larger burden due to musculoskeletal changes in cases of hyperthyroidism magnesium levels are lower and there is a preferential increase of triiodothyronine. This increase is tailored to regenerate slow twitch muscles [210]. In patients with hyperthyroidism the T3/T4 ratio is indeed altered [240] and hyperthyroid patients with a high T3/T4 ratio are prone to have a relapse [241]. Iodine metabolism in these patients is increased [242]. At a final stage the lack of magnesium could affect the function of Complex V of OXPHOS causing symptoms that have been traditionally assigned to thyroid dysfunction.

### 4.8 A comment on animal models of thyroid disease

For many years the model of active immunization with thyroglobulin has been considered to show a relation to human disease. These historical experiments aimed at the induction of thyroglobulin antibodies using organ extracts from different species together with adjuvants such as toluene, heat-killed acid fast bacteria (Dr. Jules Freund, NY), melted lanolin, mineral oil (Bayol F^2^, Esso Refining Co.), and a monooleate of manitol (Arlacel, Atlas Powder Co. ®) [243]. Another model that has been investigated in the past is that of thyroiditis in the obese strain of chickens. Following the original description of iodination of casein [244] a similar product (Protamone ®) was used in the basic diet of these birds. This casein preparation aimed at introducing a series of active thyroid hormone components. Within the extensive description of the patent for this product one can find the statement on the need to have magnesium included in order to increase the metabolic rate (e.g. paragraph 053 in [245]). This aspect is seldom mentioned later in publications dealing with this model. We found no publication dealing with magnesium and the obese strain of chickens. In the field of poultry research Hajj and Sell described the need of magnesium in order to maintain oxidative phosphorylation in the laying hen [246]. Still other authors have shown the beneficial effect of magnesium aspartate supplementation on the levels of thyroid hormones in broilers [247]. These authors even suggested the possibility of using magnesium supplementation in the obese/obese model. This suggestion was never explored. We had the opportunity to examine a small series of the last available animals of the obese strain of chickens at the Medical University of Innsbruck. This evaluation was done together with Dr. H. Dietrich and Dr. H. Talasz (Innsbruck) and Prof. Lutz Schomburg (Berlin). The chickens presented high levels of selenium in blood while liver selenoproteins were low. This picture suggests a defect in selenium utilization or transport (unpublished data from 2007) as well as a potential defect in selenoprotein synthesis. Alterations in selenium economy can be associated with changes in the immune system [248], [249]. In addition to this a modulation of gene expression profiles of Complex I, IV and V has been observed following administration of selenium [250].

We firmly believe that the central requirement for an animal model that attempts to reproduce our findings should be to include the characteristic of bipedalism; otherwise the interaction of the gastrocnemius and soleus muscles in their anti-gravitational function after having had a supination lesion will not be present. Due to similarities of the shank muscles in primates with bipedal postures similar alterations could be produced and have a close resemblance to our model [251]. Generally speaking, however, hyperthyroidism has not been described in great apes [252]. In any case one should not forget that the general transferability of animal experiments has to be taken with caution [253]. Clinical research of human conditions is still the golden standard for us.

### 4.9 A comment on the uncertainty of clinical research

Our study describes a new examination and treatment approach as well as a new explanation of benign, non-nodular thyroid disease. Although muscular changes have been observed in some cases of patients with thyroid disease, similar data that relate these events to magnesium cannot be found in the literature. The same applies to the role of psychological stress. This maybe uneasy situation will awaken the philosophical urge of the critical reader to either accept or reject our hypothesis. For this reason we will add here some notions encountered in literature that deal with the philosophy of research.

The first setting relates to the Polish School of Philosophy of Medicine which was founded by Tytus Chałubiński (1820–1889).^1^ Paraphrasing Stempsey's description [254] of the central approach taken by Chałubiński one can extract that he “proposed a method of finding proper therapy based on a holistic view of medicine and a vision of disease as disturbed function.” In our study the disturbed function is found in the musculoskeletal system. A second critical look at our results is to ask whether the methodology is plausible. We believe that modern science where genetics and “omics” are seen as indispensable tools, the simple biomechanical function will be overlooked. This is intrinsically related to the process of medical search of the most adequate method. Again in the words of Stempsey [255]: “Clinicians use many strategies and heuristics in order to formulate and test hypotheses and to draw inferences from myriad clinical data”. It follows that research will only achieve an approximation to the truth of disease. The complex situation of causality can also be read in the paper from Barata [256]: “O estudo da causalidade, portanto, diz respeito às relações de causa e efeito entre fenômenos”. This statement puts the investigation of causality into the setting of a relation between cause and effect. In our study patients with musculoskeletal changes have low levels of magnesium and a similar situation applies to psychological stressors. Finally, in a modern comment on the original work of Popper [257], Banegas et al. [258] contribute the following statement: “What we actually do is to propose a hypothesis as a tentative solution to a problem, to confront the prediction deduced from the hypothesis with actual experience, and evaluate whether the hypothesis is rejected or not by the facts.”

Considering these philosophical statements as requirements we believe that our study is within these lines as it describes mechanisms of disturbed function as well as the therapeutical approach to treat them and demonstrating clear effects on thyroid vascularization.

Study design and interpretation is an issue of lively debate. We would like to add the following quote from Albert Szent-Györgyi which we believe reflects the essence and value of observational studies: “Discovery consists of seeing what everybody has seen and thinking what nobody has thought”.^2^

## 5 Conclusions

Our observational study has shown that both thyroid vascularity as well as TSH levels are related to magnesium levels in blood. In turn low magnesium levels are related to musculoskeletal changes as well as to psychological stressors. These stressors show also a relationship to vascularity. The quantitative 3D power Doppler sonography method allows the differentiation of perfusion quality and quantity in the thyroid gland and is essential for monitoring the effects of supplementation. The novel endocrine effect of magnesium on TSH could occur by improving iodine uptake, which is an energy-dependent process.

Clinical symptoms of patients with thyroid disease can improve after correction these three central elements: the musculoskeletal changes, the effects of psychological stress, and the condition of magnesium and selenium deficiency. Since the patients described here developed signs of thyroid disease in adult age this condition can only be the result of an acquired defect. We propose that the most likely site of interaction is on Complex V of oxidative phosphorylation. In some cases coenzyme Q10 deficiency can also play a role. Supplementation with magnesium citrate and coenzyme Q10 appear to be beneficial in reversing vascular changes.

**Fig. 13:**
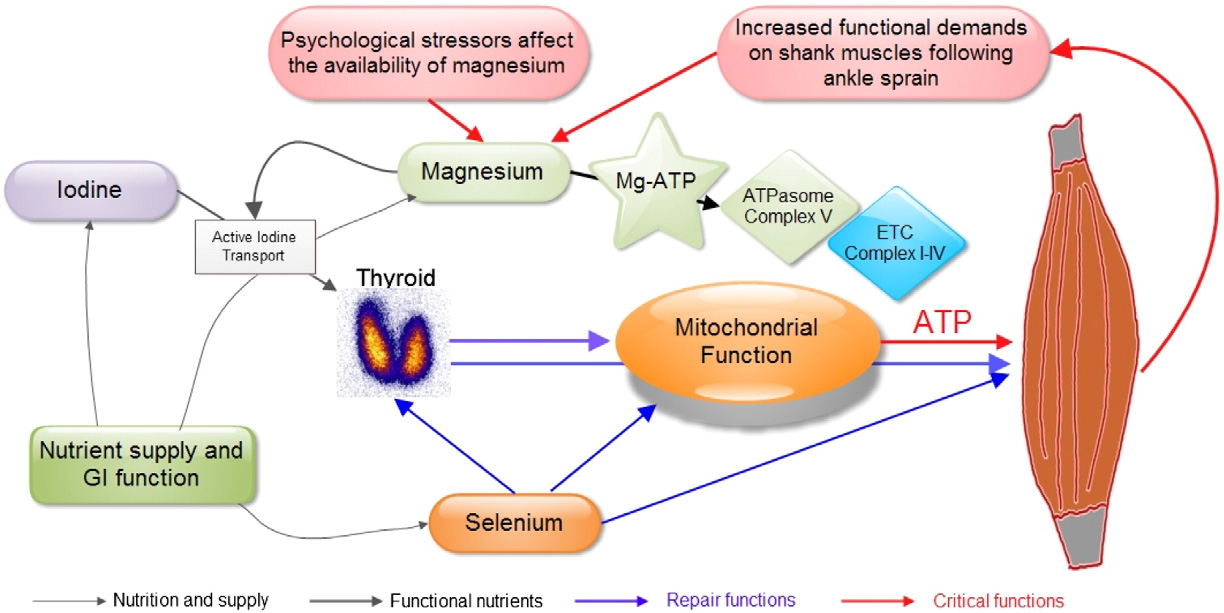
The basic elements iodine, selenium and magnesium are provided by an adequate nutrition and a healthy functioning GI tract. Magnesium is directly related to magnesium–ATP in Complex V of the ATP-some in the mitochondria. With sufficient magnesium supply the mitochondria supply enough ATP and energy-dependent processes such as iodine uptake are maintained. Selenium and selenoproteins play an important role in antioxidant functions in the body. Through the influence of physical and psychological stressors the balance of magnesium availability can be compromised leading to diminished ATP production. Physical stress augments according to the intensity of physical activity. Thyroid hormones stimulate mitochondrial biogenesis as well as muscle repair. The final clinical settings are: 1) Hypothyroidism: low magnesium levels with low iodine uptake and low need of muscle repair. 2) Hyperthyroidism: low magnesium with inefficient OXPHOS, increased need of muscle repair due to musculoskeletal changes (lateral tension, IMT) which leads to increased production of thyroid hormones triiodothyronine (preferentially) and thyroxine.

Since both hypo- and hyperthyroidism share the same selenium and magnesium deficiencies we believe that the differentiating condition leading to hyperthyroidism is that of a greater musculoskeletal alteration. The final clinical settings would be: 1) Hypothyroidism: low magnesium levels with low iodine uptake and low need of muscle repair. 2) Hyperthyroidism: low magnesium together with inefficient OXPHOS and an increased need of muscle repair due to pronounced musculoskeletal changes such as lateral tension and IMT. This condition leads to increased production of thyroid hormones triiodothyronine (preferentially) and thyroxine in order to induce muscle repair. Fig. 13 presents the graphical summary of our study.

## Abbreviations

VI: vascularization index
FI: flow index
VFI: vascularization and flow index
TRPM: transient receptor potential melastatin
IVF: in vitro-fertilization
TAO: thyroid associated ophthalmopathy
AK: Applied Kinesiology
TCM: Traditional Chinese Medicine
OXPHOS: oxidative phosphorylation

## Disclosures

RM and HM work as a team. Neither RM nor HM has any commercial association related to this study. This also applies to any non-commercial associations.

## Author contributions

RM and HM designed and carried out the clinical project. The manuscript was written by RM. Both authors approved the final manuscript. Art work was done by RM. All the diagnostic and therapeutic methods mentioned above constitute the medical philosophy of integrative medicine which we apply at our Institution. For this reason we use the term “WOMED model” in our publications.

## Funding sources

Financial support was provided exclusively by WOMED.

## Highlights: What was already known on this topic?

• Obscure origin of benign non-nodular thyroid disease.
• Some functional relationships between the musculoskeletal system and the thyroid.
• Magnesium is required for iodine uptake in the thyroid.

## What this study adds

• Definition of normal levels of magnesium in normal subjects as > 0.95 mmol/l.
• Normalization of TSH levels after Mg supplementation.
• 3D-perfusion patterns of the thyroid are responsive to supplementation with magnesium citrate and in some cases together with coenzyme Q10.
• Reversibility of morphological changes in the thyroid after supplementation.
• Description of additional conditions associated with increased thyroid perfusion such as psychological stress, physical stress, infection.
• Need to treat psychological and physical stress in order to reduce magnesium needs.
• Increased thyroid perfusion in the first days after in-vitro fertilization and egg transfer.
• Potential of detecting cases of coenzyme Q10 deficiency based on the vascularization index and relation of vascularity to oral contraceptives.
• The WOMED model as holistic clinical tool than can be applied successfully.

## Figures and Tables

**Table 1:** Changes in TSH and fT4 levels given in μU/ml after exclusive supplementation with magnesium citrate at a dose of 3 × 1.4 mmol/d.

												mean ± S.D.
TSH t0	4	4.6	3.9	8.1	5.7	2.3	6.1	6.3	21	17.3	5.1	7.6 ± 5.9
TSH t1	2.1	3.2	1.6	3.47	1.5	1.6	3.1	3	4	3.1	2.7	2.67 ± 0.8
fT4 t0	15.1	18.3	16.1	15.6	18.6	18.4	10.9	9.1	8.8	10.4	13.4	14.06 ± 3.7
fT4 t1	15.4	14.8	14.4	11.6	16.7	15.3	17.4	11.1	14.2	14.5	14.1	14.5 ± 1.8

## Appendix A Supplementary data

Supplementary video Demonstration of the idiopathic moving toes finding.

## Footnotes

1 “Starsza” polska szkoła filozofii medycyny http://pl.wikipedia.org/wiki/Polska_szko%C5%82a_filozofii_medycyny.

2 http://thinkexist.com/quotes/albert_szent-gyorgyi/.
