# Supplementary figures and images for: The WOMED model of benign thyroid disease: Acquired magnesium deficiency due to physical and psychological stressors relates to dysfunction of oxidative phosphorylation
Source: BBA Clin. 2014 Nov 12;3:44–64. doi: 10.1016/j.bbacli.2014.11.002 (PMC4661500; doi:10.1016/j.bbacli.2014.11.002)

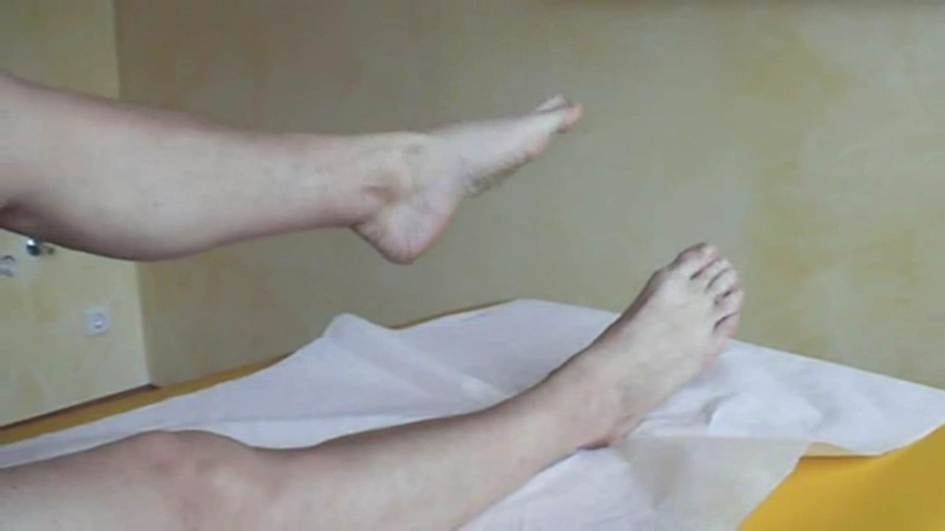

Supplement: Supplementary video — Demonstration of the idiopathic moving toes finding. [file mmc1.jpg]
